# Species-specific IL-1β is an inflammatory sensor of Seneca Valley Virus 3C Protease

**DOI:** 10.1371/journal.ppat.1012398

**Published:** 2024-07-22

**Authors:** Xiangyu Huang, Zhenchao Zhao, Cheng Zhu, Lvye Chai, Ya Yan, Ye Yuan, Lei Wu, Minjie Li, Xiaohan Jiang, Haiwei Wang, Zheng Liu, Pingwei Li, Xin Li

**Affiliations:** 1 National Key Laboratory of Veterinary Public Health and Safety, College of Veterinary Medicine, China Agricultural University, Beijing, China; 2 Key Laboratory of Animal Epidemiology of the Ministry of Agriculture and Rural Affairs, College of Veterinary Medicine, China Agricultural University, Beijing, China; 3 Tianjin Key Laboratory of Function and Application of Biological Macromolecular Structures, School of Life Sciences, Tianjin University, Tianjin, China; 4 State Key Laboratory of Animal Disease Control, Harbin Veterinary Research Institute, Chinese Academy of Agricultural Sciences, Harbin, China; 5 Koblika Institute of Innovative Drug Discovery, School of Medicine, Chinese University of Hong Kong, Shenzhen, Guangdong, China; 6 Department of Biochemistry and Biophysics, Texas A&M University, College Station, Texas, United States of America; Stanford University, UNITED STATES OF AMERICA

## Abstract

Inflammasomes play pivotal roles in inflammation by processing and promoting the secretion of IL-1β. Caspase-1 is involved in the maturation of IL-1β and IL-18, while human caspase-4 specifically processes IL-18. Recent structural studies of caspase-4 bound to Pro-IL-18 reveal the molecular basis of Pro-IL-18 activation by caspase-4. However, the mechanism of caspase-1 processing of pro-IL-1β and other IL-1β-converting enzymes remains elusive. Here, we observed that swine Pro-IL-1β (sPro-IL-1β) exists as an oligomeric precursor unlike monomeric human Pro-IL-1β (hPro-IL-1β). Interestingly, Seneca Valley Virus (SVV) 3C protease cleaves sPro-IL-1β to produce mature IL-1β, while it cleaves hPro-IL-1β but does not produce mature IL-1β in a specific manner. When the inflammasome is blocked, SVV 3C continues to activate IL-1β through direct cleavage in porcine alveolar macrophages (PAMs). Through molecular modeling and mutagenesis studies, we discovered that the pro-domain of sPro-IL-1β serves as an ’exosite’ with its hydrophobic residues docking into a positively charged 3C protease pocket, thereby directing the substrate to the active site. The cleavage of sPro-IL-1β generates a monomeric and active form of IL-1β, initiating the downstream signaling. Thus, these studies provide IL-1β is an inflammatory sensor that directly detects viral protease through an independent pathway operating in parallel with host inflammasomes.

## Author summary

IL-1β is a key inflammatory mediator, crucial for immune responses and pathological inflammation. Upon the assembly of large complexes by NOD-like receptors, caspase-1 serves as the endogenous enzyme that cleaves Pro-IL-1β in activate IL-1β. Previous studies have identified IL-1β is an innate immune sensor of group A Streptococcus (GAS) protease SpeB. However, whether IL-1β is an inflammatory sensor for viral proteases remains unclear. Here, we report that Seneca Valley Virus (SVV) 3C protease functions as an unconventional IL-1β-converting enzyme (ICE), specifically and directly cleaving and processing swine Pro-IL-1β but not human Pro-IL-1β. The cleaved IL-1β is bioactive, consistent with the function of mature IL-1β. This finding reveals a novel virus-mediated activation mechanism of IL-1β and may influence the host’s inflammatory response.

## Introduction

NOD-like receptors (NLRs) detect viral invasions by sensing pathogen-associated molecular patterns or danger signals and activate caspase-1 via the canonical inflammasomes. Upon activation, caspase-1 cleaves Pro-IL-1β and Pro-IL-18, which exist as inactive cytoplasmic precursors and their N-terminal domain needs be cleaved to be biologically active [1]. Once released into the extracellular space from living cells [2,3] or through a lytic form of cell death (referred to as pyroptosis [4–7]), IL-1β and IL-18 bind to inflammation-inducing receptors on adjacent cells, inducing a potent inflammatory response [1]. Consequently, the regulation of IL-1β and IL-18 maturation is garnering increasing interest due to its critical roles in innate immunity. Recently, the structures of caspase-4 in complex with Pro-IL-18 have been determined [8,9], providing important insights into the mechanism of Pro-IL-18 activation by caspase-4. However, the molecular mechanisms of Pro-IL-1β maturation by caspase-1 or other prototypical IL-1β-converting enzymes (ICE) remain to be defined.

The activation of IL-1β by caspase-1 is tightly controlled by two-tiered regulation system, which includes NLRP3 activation and auto-proteolytic processing of caspase-1 [10]. Aside from precise regulation by caspase-1, pathogenic bacterial enzymes such as SpeB and LasB directly cleaves Pro-IL-1β in a bioactive IL-1β that can cause host inflammation-related diseases [11–13]. However, it is not known if viral enzymes can serve as an ICE that cleaves Pro-IL-1β. The molecular mechanisms of Pro-IL-1β cleavage and activation by ICE are also not so clear. In this study, we observed that picornavirus SVV 3C protease is a novel ICE, selectively cleaving the oligomeric precursor of swine Pro-IL-1β and generates mature monomeric IL-1β (cIL-1β). This cleaved IL-1β is biologically active and prompts porcine alveolar macrophages (PAMs) to initiate a strong inflammatory response, thereby inhibiting viral replication.

## Results

### SVV infection induces high level expression of IL-1β

**Fig 1:**
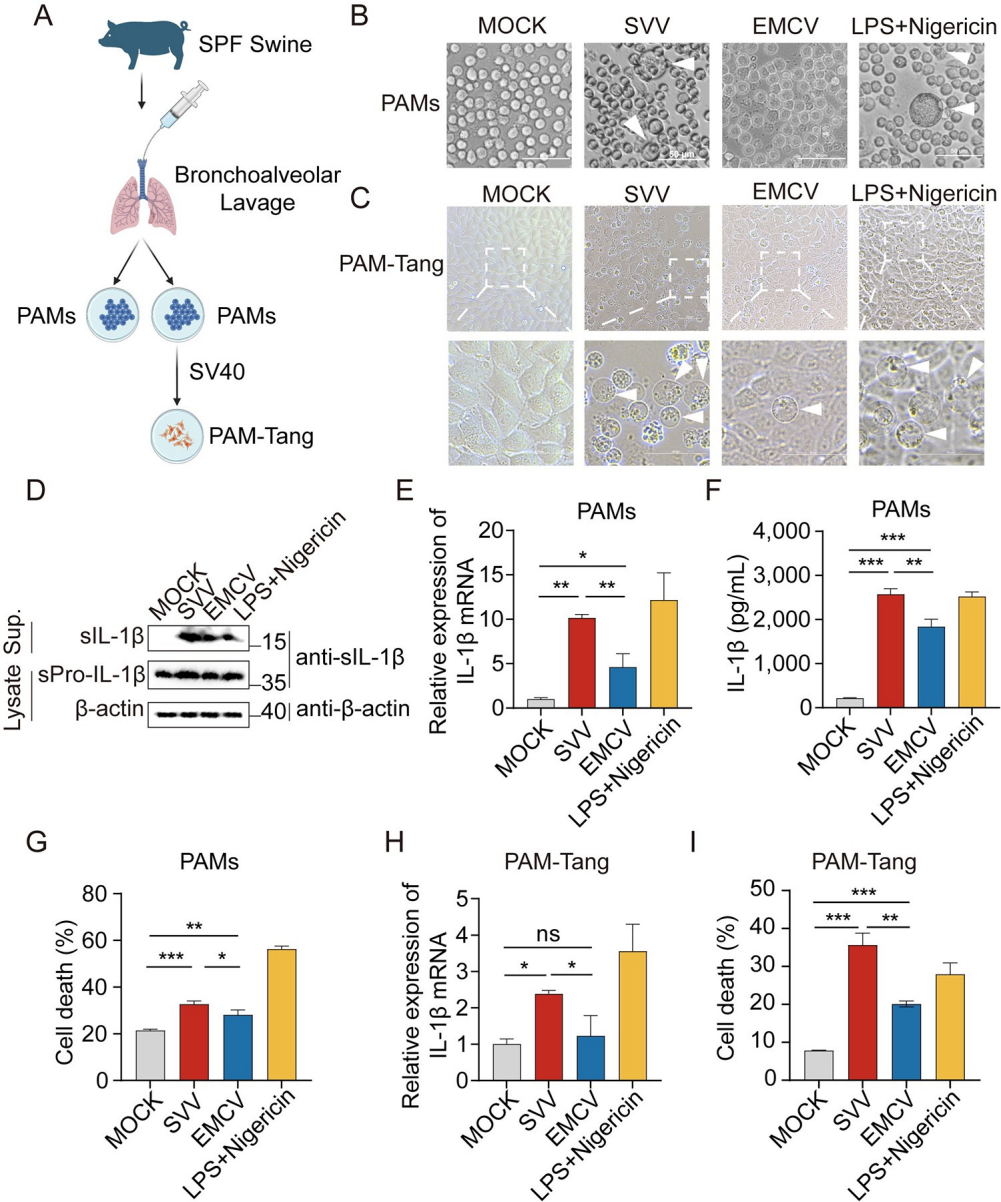
SVV infection induces high level expression of IL-1β. (**A**) Illustration of the preparation process for PAMs and PAM-Tang cells. Illustration was created with Biorender. B to I, PAMs or PAM-Tang cells were infected with SVV and EMCV at MOI of 10 for 12 hours, respectively. (**B** and **C**) The cell morphology of PAMs (**B**) and PAM-Tang (**C**) was observed using an Evos FL Auto2 fluorescence microscope (white arrows, pyroptotic cells). Bars = 50 μm. (**D**) Mature sIL-1β, sPro-IL-1β and β-actin protein were determined by Western Blotting. (**E** and **H**), Swine IL-1β mRNA levels (related to swine GAPDH) were analyzed by quantitative RT-PCR (qPCR). (**F**) Swine IL-1β levels in the cell culture supernatants were detected by ELISA. (**G** and **I**) LDH-release-based cell death measurement in the cell culture supernatants were detected by LDH assay kit. PAMs and PAM-Tang cells were treated with LPS for 8 hours and with nigericin for 4 hours as controls. A *P* value of less than 0.05 was considered statistically significant. * for *P*<0.05, ** for *P*<0.01, *** for *P*<0.001, ns for no significant.

Swine are susceptible to SVV and encephalomyocarditis Virus (EMCV) infection, presenting symptoms such as pulmonary edema, congestion, and infiltration by inflammatory cells [14–17]. To assess the level of inflammatory cytokine secretion following infection by SVV and EMCV, PAMs and PAM-Tang cells[18] (primary immortalized PAMs) (Fig 1A) were infected with SVV and EMCV at MOI (Multiplicity of Infection) of 10 for 12 h, using lipopolysaccharides (LPS) and nigericin as positive controls for stimulation. Cells infected with SVV underwent noticeable morphological alterations indicative of pyroptosis, such as cell rounding, swelling, and a distinct “fried-egg” appearance due to nucleus protrusion, akin to the effects observed in the LPS and nigericin stimulated cells. By contrast, PAMs infected with EMCV do not exhibit the significant morphological changes (Fig 1B and 1C). Immunoblot analysis revealed the presence of swine IL-1β (sIL-1β) in the supernatant of PAMs infected by both SVV and EMCV (Fig 1D). Notably, the mRNA and protein levels of sIL-1β were markedly elevated in the SVV-infected group as compared to the EMCV group (Fig 1E and 1F). In line with these observations, the release of lactate dehydrogenase (LDH), a marker of lytic cell death, was substantially increased in PAMs infected with SVV, indicating a higher rate of cell death (Fig 1G). Furthermore, SVV infection prompted an enhanced expression of sIL-1β and the release of LDH in PAM-Tang cells (Fig 1H and 1I). Collectively, these results show that SVV infection elicits a more pronounced inflammatory response than EMCV infection in both PAMs and PAM-Tang cells.

### SVV 3C protease is a novel IL-1β-converting enzyme

**Fig 2:**
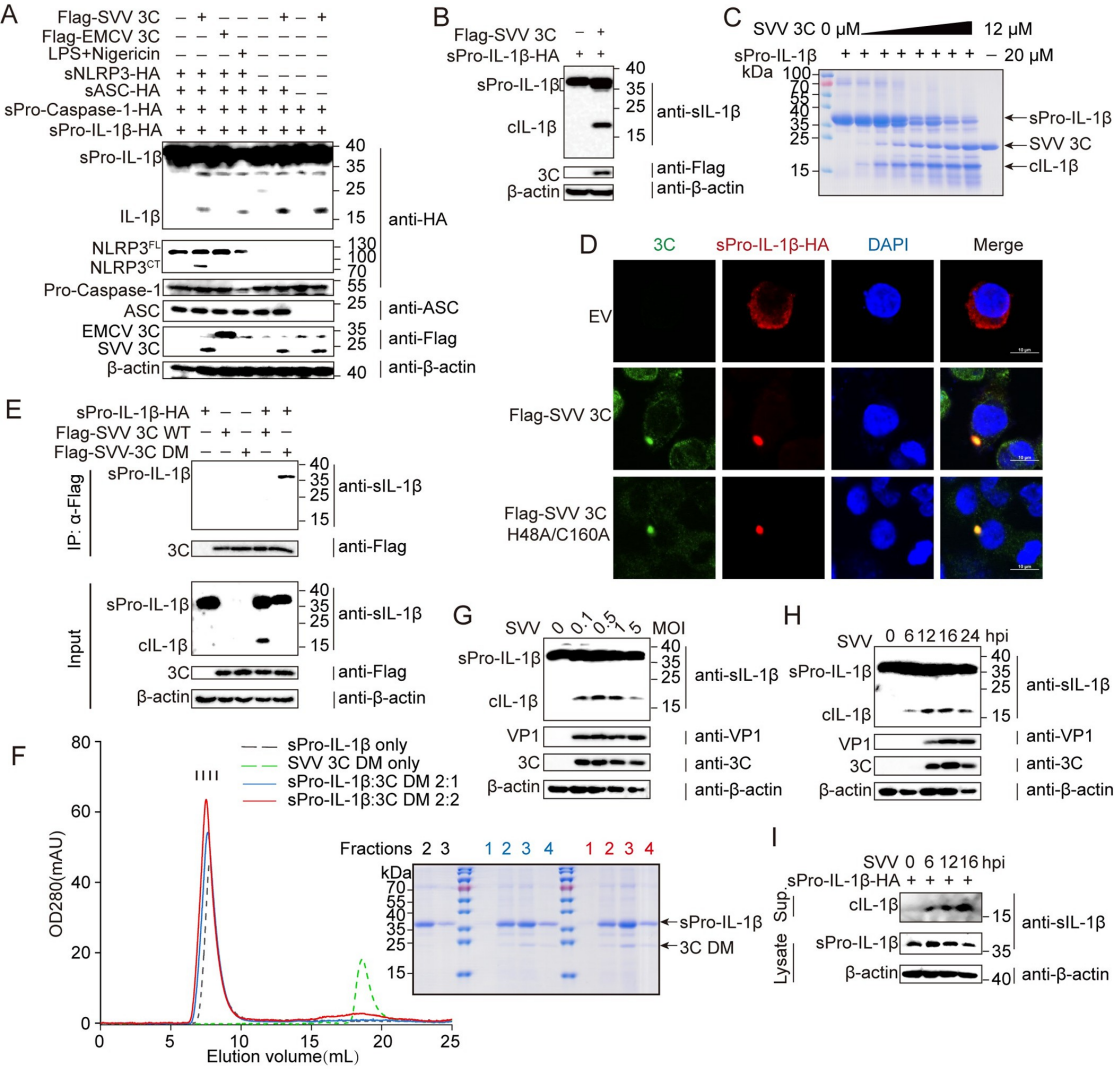
SVV 3C protease is a novel IL-1β-converting enzyme. (**A**) HEK-293T cells were transfected with plasmids encoding Flag-SVV 3C or Flag-EMCV 3C in the presence of sNLRP3 inflammasome system or system lacking sNLRP3-HA, sASC-HA, sPro-caspase-1-HA. LPS and nigericin were added after transfection for 12 hours as controls. (**B**) HEK-293T cells were cotransfected with plasmids encoding sPro-IL-1β and SVV 3C. (**C**) SDS-PAGE analysis of in vitro cleavage of sPro-IL-1β in a reaction buffer containing 25 μM sPro-IL-1β recombinant protein with different dose of purified recombinant protein SVV 3C (0.25–12 μM) for 2 h at 37°C. (**D**) Confocal microscopy analysis of colocalization between sPro-IL-1β and SVV 3C. HEK293T cells were cotransfected with plasmids encoding sPro-IL-1β and Flag-wild type 3C or Flag-H48A/C160A 3C. Bars = 10 μm. (**E**) HEK-293T cells were cotransfected with plasmids encoding sPro-IL-1β and Flag-wild type 3C or Flag-H48A/C160A. (**F**) Binding studies of sPro-IL-1β with 3C DM (H48A/C160A) by gel filtration chromatography. Elution profile of sPro-IL-1β is in black and the 3C DM in green. Elution profiles of mixtures of sPro-IL-1β and 3C DM at molar ratios 2:1 and 2:2 are in blue and red, respectively. (**G** and **H**) HEK-293T cell line stably expressing sPro-IL-1β were infected with SVV at various MOIs as indicted for 16 h (**G**) or infected with SVV at an MOI of 0.1 at the indicated times (**H**). (**I**) PAM-Tang cells were transfected with sPro-IL-1β and then infected with SVV at an MOI of 0.1 at the indicated times. Cell supernatants (Sup.) and lysates were analyzed by immunoblotting.

During viral infection, a complex cascade involving NLRP3, ASC and Pro-caspase-1 forms to process the precursors of IL-1β and IL-18 into active forms. While previous reports the SVV 3C protease cleaves NLRP3, causing it to become inactive [19]. However, this cannot explain why SVV infection still elicits a significantly more inflammatory response than EMCV infection. Therefore, we hypothesize that there might be alternative pathways for producing IL-1β upon SVV infection. We have investigated the potential roles of SVV 3C protease to activate the NLRP3 inflammasome using an in-vitro reconstitution system in HEK-293T cells. Although our data is consistent with the previous report that SVV 3C protease can cleave NLRP3 [19], it also cooperates with inflammasome components including NLRP3, ASC, Pro-caspase-1 and Pro-IL-1β to induce sIL-1β production, akin to established positive control (Fig 2A). Conversely, the EMCV 3C protease fails to activate the NLRP3 inflammasome as effectively as SVV 3C. As parallel controls, we set up the reconstitution of SVV 3C with NLRP3 inflammasome lacking each component. Interestingly, we observed that sIL-1β was still generated in the reconstitution assay in the absence of NLRP3, ASC, or Pro-caspase-1 (Fig 2A). Given the proteolytic activity of the SVV 3C protease, we hypothesized that it is likely that SVV 3C directly cleave Pro-IL-1β to produce active IL-1β independent of the NLRP3 inflammasome. This hypothesis was tested by co-transfecting HEK-293T cells with plasmids encoding Flag-SVV 3C and sPro-IL-1β-HA. Subsequent immunoblotting confirmed the presence of cleaved IL-1β (cIL-1β) of approximately 17 kDa in size, indicting the direct cleavage of IL-1β (Fig 2B). To test if this cleavage of sPro-IL-1β by SVV 3C is direct, we purified sPro-IL-1β and SVV 3C proteins by gel filtration chromatography followed by ion exchange chromatography (S1A and S1B Fig). When recombinant SVV 3C was incubated with sPro-IL-1β, we observed a dose-dependent decrease of the sPro-IL-1β band coupled to the emergence of a new band of approximately 17 kDa (Fig 2C), a result corroborated by immunoblotting (S2A Fig). This contrasted with co-expression experiments involving sPro-IL-1β and EMCV 3C, which did not yield detectable amount of cleaved IL-1β (S2B Fig). Furthermore, hPro-IL-1β cannot be cleaved by SVV 3C to produce mature IL-1β both in cell and *in vitro*. (S1C, S2C and S2D Figs). While caspase-1 is known to cleave most IL-1-family cytokines, such as Pro-IL-1β, Pro-IL-18 and Pro-IL-33, we did not observe the cleavage of sPro-IL-18-HA and sPro-IL-33-HA when co-expressed with SVV 3C in HEK293T cells, indicating a specific cleavage activity for sPro-IL-1β (S2E and S2F Fig). *Picornaviridae* is a large family of RNA viruses, including SVV, EMCV, EV-A71, HAV, and PV. To investigate if other picornaviruses 3C also cleave Pro-IL-1β, we co-expressed FLAG-tagged EV71 3C, HAV 3C, PV-1 3C, and PV-3 3C along with hPro-IL-1β in HEK-293T cells. Surprisingly, we did not detect the cleavage of hPro-IL-1β by these 3C proteases (S3A Fig). Previous studies have reported that various viruses possess protease-like proteins. To determine whether other viral proteases also cleave Pro-IL-1β, we co-expressed FLAG-tagged SARS-CoV-2-NSP5, PDCoV-NSP5, PEDV-NSP5, ASFV-pS273R, PRRSV-NSP4 and PRRSV-NSP2 along with either hPro-IL-1β or sPro-IL-1β. Similarly, these proteases did not cleave Pro-IL-1β in the transfected cells (S3B and S3C Fig).

To test if SVV 3C interacts with sPro-IL-1β, HEK-293T cells were transfected with sPro-IL-1β and either SVV 3C WT or 3C H48A/C160A (DM, double mutation). We performed confocal microscopy and co-immunoprecipitation (Co-IP) to analyze their interaction. The microscopic co-localization analysis showed that both SVV 3C WT and 3C H48A/C160A co-localized with sPro-IL-1β in the cytoplasm (Fig 2D). The Co-IP results also indicated that 3C H48A/C160A co-immunoprecipitated with sPro-IL-1β (Fig 2E). Previous studies have reported that SVV 3C WT mediates a high-speed dynamic enzymatic reaction, resulting the Co-IP band between 3C WT and Pro-IL-1β is much weaker [20,21]. Gel filtration chromatography also demonstrated direct binding between recombinant sPro-IL-1β and SVV 3C protein as indicated by a shift in peak at two binding ratios which was further verified by SDS-PAGE (Figs 2F, S4A and S4B). To contextualize within a viral infection environment, we explored whether SVV infection prompts the release of cIL-1β independent of caspase activity. To exclude the potential contributions of gasdermin (GSDM) proteins, which are known to facilitate mature IL-1β release, we used a HEK293T cells cell line stably expressing sPro-IL-1β and lacking GSDM proteins using lentiviral over-expression system (S3D Fig). To determine whether sPro-IL-1β was cleaved during infection, we challenged HEK-293T cells stably expressing sPro-IL-1β with SVV (MOI = 0.1, 0.5, 1, and 5), and collected the cells at 16 h post-infection. The results showed that bands of ~17 kDa appeared at the bottom of the gel after 16 h of infection at different MOIs, indicating the presence of cIL-1β (Fig 2G). To confirm these results, HEK-293T cells stably expressing sPro-IL-1β were challenged with SVV (MOI = 0.1) at 6, 12, 16 and 24 h post-infection. The results showed an increase of cIL-1β levels at 6 h, 12 h and 16 h post-infection, with a reduction at 24h (Fig 2H), suggesting that SVV infection causes sIL-1β cleavage to produce active cIL-1β. The biological function of IL-1β is initiated upon its secretion into the extracellular region. To determine whether cIL-1β could be secreted following cleavage, PAM-Tang cells were transfected with sPro-IL-1β and then infected with SVV at an MOI of 0.1 at 6 h, 12 h, 16 h and 24 h. Immunoblotting showed that cIL-1β expressed in the cell supernatant, reaching its peak at 16 hpi (Fig 2I). Taken together, our data suggests that the direct cleavage of sPro-IL-1β by SVV 3C, which is unique among viral proteases.

### SVV 3C protease cleaves sPro-IL-1β at a “LQ” motif

**Fig 3:**
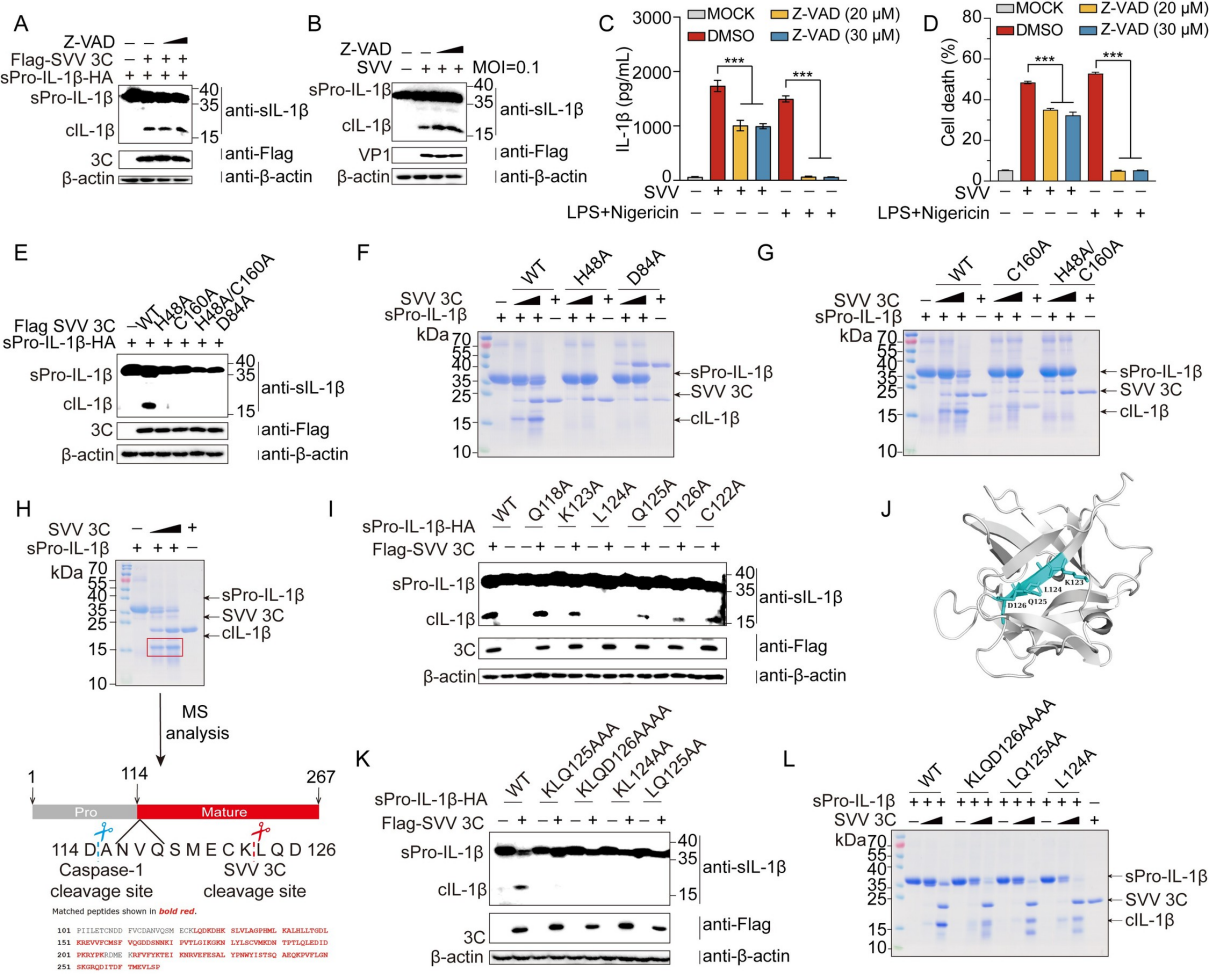
SVV 3C protease cleaved sPro-IL-1β at a “LQ” motif. (**A**, **B**, **C** and **D**) Effects of Z-VAD-FMK on cleavage induced by SVV 3C. (**A**) HEK293T cells were cotransfected with plasmids encoding sPro-IL-1β and SVV 3C with or without Z-VAD-FMK (20 μM or 30 μM). (**B**) HEK-293T cell line stably expressing sPro-IL-1β were infected with SVV at an MOI of 0.1 for 16 h with or without Z-VAD-FMK (20 μM or 30 μM). (**C** and **D**) PAMs were infected with SVV or stimulated with LPS and Nigericin in the presence or absence of Z-VAD-FMK (20 μM or 30 μM). The release of IL-1β (**C**) and LDH (**D**) in the cell supernatant was measured. A *P* value of less than 0.05 was considered statistically significant. *** for *P*<0.001. (**E**, **F** and **G**) Catalytic residues of 3C protease active sites effects on sPro-IL-1β cleavage. (**E**) HEK-293T cells were cotransfected with plasmids encoding sPro-IL-1β and 3C or its catalytic residues mutants for 24 h. (**F** and **G**) SDS-PAGE analysis of *in vitro* cleavage of 25 μM sPro-IL-1β recombinant protein with different dose of purified recombinant protein wild-type or mutant SVV 3C (WT, H48A, D84A, C160A, H48A/C160A) for 2 h at 37°C. (**H**) Cleaved sPro-IL-1β band (marked by red box) for mass spectrometry analysis. (**I**, **J**, **K** and **L**) Cleavage sites of sPro-IL-1β induced by SVV 3C. (**I** and **K**), HEK-293T cells were cotransfected with plasmids encoding SVV 3C and sPro-IL-1β or its mutants (Q118A, C122A, K123A, L124A, Q125A, D126A, KL124AA, LQ125AA, KLQ125AAA, KLQD126AAAA). (**J**), The K123, L124, Q125, D126 are displayed in the modeling structure of sPro-IL-1β using PyMOL software. (**L**), SDS-PAGE analysis of *in vitro* cleavage of 25 μM sPro-IL-1β recombinant protein or its mutants (L124A, LQ125AA and KLQD126AAAA) recombinant protein with different dose of purified recombinant protein SVV 3C for 2 h at 37°C.

To investigate whether 3C cleave sPro-IL-1β is caspase-dependent, HEK-293T cells were co-transfected with SVV 3C and sPro-IL-1β in the presence of pan-caspase inhibitor, Z-VAD-FMK. The results showed that Z-VAD had no inhibitory effects on the cleavage of sPro-IL-1β by SVV 3C (Fig 3A). To further determine whether SVV infection induced the cleavage of sPro-IL-1β is caspase-independent, HEK-293T cells stably expressing sPro-IL-1β were treated with Z-VAD, followed by SVV infection (MOI = 0.1) for 16 h. The results indicated that, in the presence and absence of Z-VAD, SVV infection resulted in sPro-IL-1β cleavage, leading to the production of cIL-1β (Fig 3B). To provide a physiological context, primary porcine alveolar macrophages (PAMs), obtained from SPF (specific pathogen free) pigs through alveolar lavage, are key in studying porcine immunology due to their biological relevance and physiological accuracy. After treatment with Z-VAD, PAMs were either infected with SVV for 12 hours or stimulated with LPS and Nigericin. The results indicated that Z-VAD treatment completely inhibited the release of IL-1β and LDH induced by LPS and Nigericin stimulation, but only partially inhibited the release of IL-1β and LDH caused by SVV infection (Fig 3C and 3D). This suggests that the release of IL-1β and LDH during SVV infection can occur independently of inflammasome. The conserved catalytic residues of SVV 3C include H48, D84, and C160, which are critical for the enzymatic activity [21]. To test whether the catalytic activity of 3C protease is critical for the processing of sPro-IL-1β, residues H48, D84, C160, and H48/C160 were mutated to alanine. Subsequently, SVV 3C WT and its catalytically inactive mutants were co-transfected with sPro-IL-1β into HEK-293T cells. We observed that 3C WT processed sPro-IL-1β, generating cIL-1β, while the four enzymatically inactive mutants were unable to process sPro-IL-1β, suggesting specific substrate recognition (Fig 3E). Moreover, when incubated with the recombinant protein sPro-IL-1β with 3C and its mutants, none of the four SVV 3C mutant proteins led to the cleavage of sPro-IL-1β to generate cIL-1β (Fig 3F and 3G), consistent with results from the cell-based experiments. The IL-1β tetrapeptide sequence YVHD116 is known to be targeted by caspase-1[22]. To determine the cleavage sites of SVV 3C on sPro-IL-1β, sPro-IL-1β was incubated with SVV 3C, and the resulting ~17 kDa cleavage product was analyzed by mass spectrometry, which revealed the first identifiable peptide segment starts from L124 (Fig 3H). To pinpoint the exact cleavage site, we substituted amino acids Q118A, C122, K123, L124, Q125, and D126 with alanine. Remarkably, mutations L124A significantly reduced SVV 3C cleavage, while K123A, Q125A and D126A mutations showed varying degrees of reduction of cleavage. The Q118A and C122A mutants were not diminished (Fig 3I). These residues were mapped onto the structure of sPro-IL-1β generated by AlphaFold (Fig 3J). In addition, we constructed multiple mutants close to L124, including KL124AA, LQ125AA, KLQ125AAA, and KLQD126AAAA. The cleavage by SVV 3C was drastically reduced in these mutants (Fig 3K). To verify these findings, we purified the recombinant L124A, LQ125AA, and KLQD126AAAA proteins (S5A–S5C Fig). The cIL-1β level was significantly reduced when these mutants of sPro-IL-1β were incubated with SVV 3C (Fig 3L). However, it does not diminish the IL-1β production by caspase-1 mediated-cleavage of these mutants (S1D and S3E Figs). Collectively, these results indicate that SVV 3C protease cleaves sPro-IL-1β at the “LQ” motif, a mechanism distinct from caspase-1 cleavage.

### A unique ‘Exosite’ is required for SVV 3C cleavage

**Fig 4:**
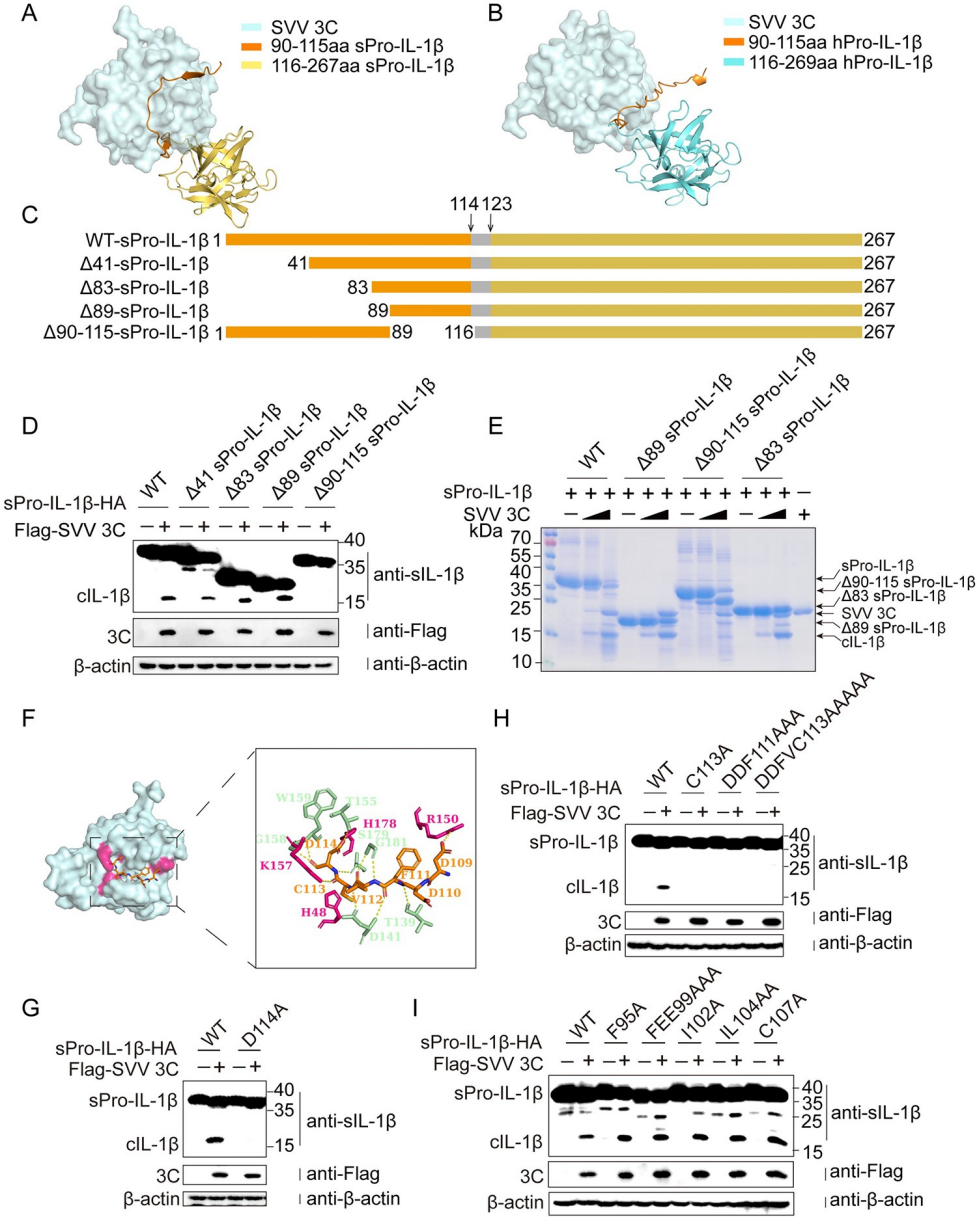
A unique ‘Exosite’ is required for SVV 3C cleavage. (**A** and **B**) Binding model of sPro-IL-1β or hPro-IL-1β with SVV 3C. The complex model of sPro-IL-1β/SVV 3C and hPro-IL-1β/SVV 3C were constructed using AlphaFold-Multimer. (**C** to **I**), The exosite of sPro-IL-1β bound by SVV 3C. (**C**) Schematic of full-length sPro-IL-1β and its four truncated mutants. (**D**) HEK-293T cells were cotransfected with plasmids encoding SVV 3C and sPro-IL-1β or its truncated (Δ41, Δ83, Δ89 and Δ90–115) for 24 h. (**E**) SDS-PAGE analysis of *in vitro* cleavage of sPro-IL-1β or its truncated (Δ89, Δ90–115 and Δ83) recombinant protein with different dose of purified recombinant protein SVV 3C for 2 h at 37°C. (**F**) Detailed interaction interface of sPro-IL-1β and SVV 3C at Exosite. Positively charged pocket of SVV 3C (H48, R150, K157, H178) was marked in hot pink and 109-114aa domain of sPro-IL-1β was marked in orange. (**G**, **H** and **I**) HEK-293T cells were cotransfected with plasmids encoding SVV 3C and sPro-IL-1β or its mutants of 90-115aa domain (F95A, FEE99A, I102A, IL104AA, C107A, C113A, DDF111AAA, DDFVC113AAAAA, D114A) for 24 h.

The cleavage of the sPro-IL-1β by SVV 3C protease requires more than just the "LQ" motif, as this sequence is conserved in human and swine Pro-IL-1β, but SVV 3C cannot cleave hPro-IL-1β to produce cIL-1β (S6A Fig). This specificity parallels the recognition of human Pro-IL-18 by human caspase-4, which cannot cleave mouse Pro-IL-18 despite their shared tetrapeptide motif LESD36. The pro-domain of human Pro-IL-18 has been identified as essential for this recognition by caspase-4 [8, 9]. We propose that a similar pro-domain of sPro-IL-1β is crucial for SVV 3C recognition. Then we modelled the complex of sPro-IL-1β-SVV 3C and hPro-IL-1β-SVV 3C using AlphaFold multimer [23]. In both the swine and human Pro-IL-1β structures, the disordered N-terminal region (residues 1–115) wrapped around SVV 3C, with possible functions as ‘exosite’. Specifically, residues 90–115 of sPro-IL-1β mediated most interface interactions (Fig 4A). However, the same residues of human Pro-IL-1β formed knotted structure covering the enzymatic site in several possible models of interactions (Fig 4B). We constructed deletion mutants of residues 1–41, 1–83, 1–89, and 90–115 of sPro-IL-1β, and these deletion mutants were co-transfected with SVV 3C in HEK-293T cells. Consistent with the model, SVV 3C still cleaved sPro-IL-1β after the deletion of residues 1–89, while the deletion of residues 90 to 115 prevented cleavage by SVV 3C (Fig 4C and 4D). Purified sPro-IL-1β proteins with the deletions of residues 1–83, 1–89, and 90–115 were co-incubated with SVV 3C (S5D–S5F Fig). Consistent with the above results, deletion of residues 1–89 of sPro-IL-1β does not affect cleavage, whereas the deletion of residues 90 to 115 prevents cleavage by SVV 3C, resulting in no production of cIL-1β (Fig 4E). Our structural model identified the sequence 109-DDFVCD-114 of sPro-IL-1β that fit into a positively charged pocket (H48, R150, K157, H178) of SVV 3C (Figs 4F and S6C), bringing the substrate sequence (124-LQ-125) close to the active site. However, in hPro-IL-1β, amino acid residues 90–115 form a knotted structure around the active site in several possible interaction models, and the segment 109-DNEAYV-114 does not interact with the 3C pocket as observed in sPro-IL-1β (S6B and S6D Fig). Given that residue D114 is the caspase-1 cleavage site, we assessed the importance of D114 in SVV 3C cleavage. The D114A mutant of sPro-IL-1β was resistant to cleavage by SVV 3C (Fig 4G). To investigate the subtle interactions between 109-DDFVC-113 of sPro-IL-1β with SVV 3C, we constructed multiple mutants likely to impact the interactions, including C113A, DDF111AAA, DDFVC113AAAAA, as a control we also constructed mutants F95A, FEE99AAA, I102A, IL104AA, C107A between residues 90 and 109 of sPro-IL-1β. Consistent with the structural models, we observed that mutation C113A, DDF111AAA, DDFVC113AAAAA, D114A prevented cleavage by SVV 3C, while mutants F95A, FEE99AAA, I102A, IL104AA, C107A can still be cleaved by SVV 3C (Fig 4H and 4I). Taken together, these results indicates that residues 109–114 in the pro-domain of sPro-IL-1β is a crucial ‘exosite’ for the recognition and cleavage by SVV 3C.

### The cleavage of IL-1β by SVV 3C generates an active cytokine

**Fig 5:**
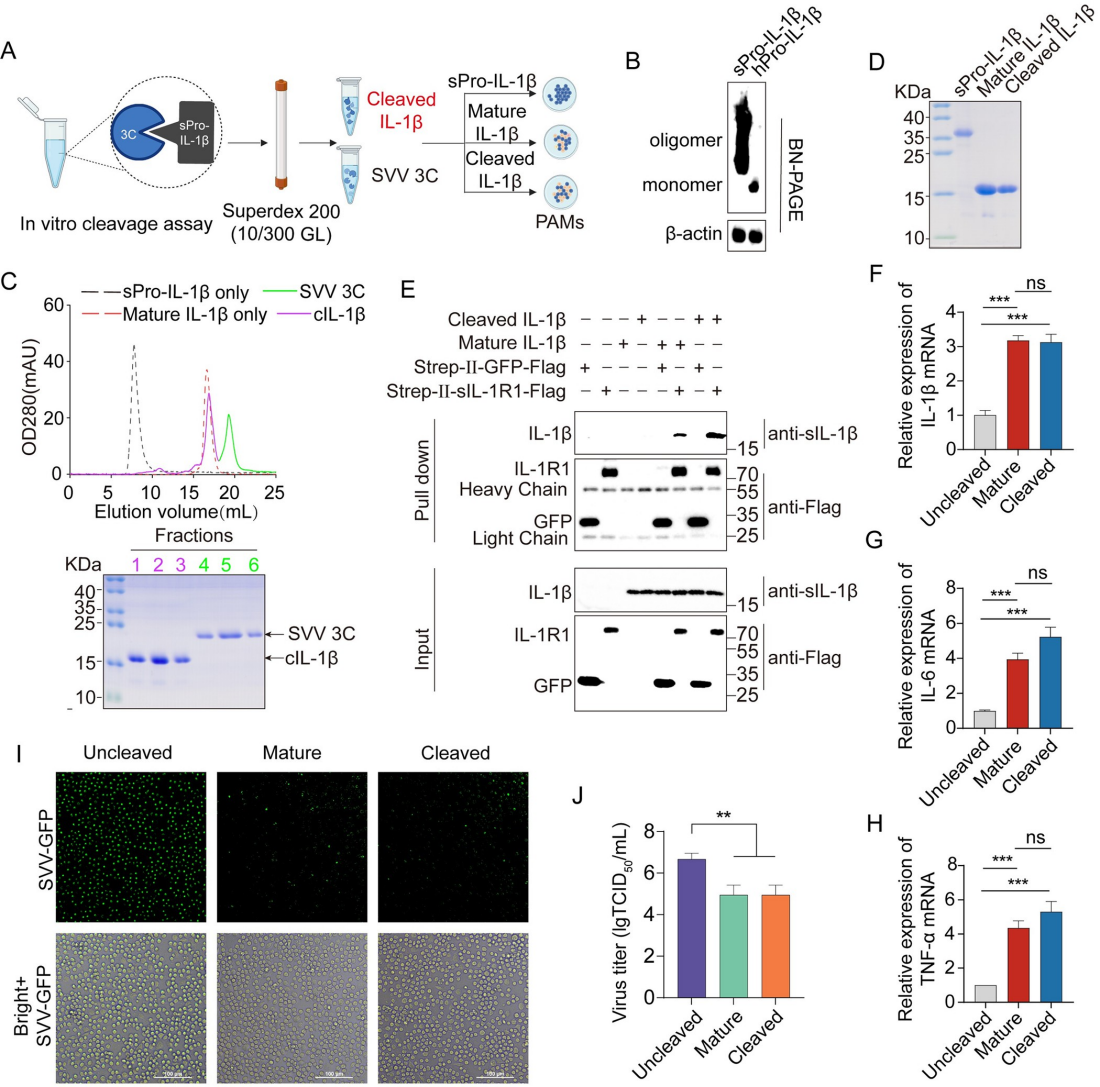
The cleavage of IL-β by SVV 3C generates an active cytokine. (**A**) Illustration of purification of cIL-1β produced by *in vitro* cleavage of sPro-IL-1β by SVV 3C. Illustration was created with Biorender. (**B**) Blue Native PAGE of HEK-293T cells were transfected with sPro-IL-1β or hPro-IL-1β. (**C** and **D**) The formation of cleavage-IL-1β protein was analyzed by gel filtration chromatography. SVV 3C was incubated with sPro-IL-1β at 37°C for 2 h, and each independent peak fraction was analyzed by SDS-PAGE (**C**). Elution profile: sPro-IL-1β (black), mature IL-1β (red), cIL-1β (pink), SVV 3C (green). (**D**) SDS-PAGE analysis of purified Pro-IL-1β, Mature IL-1β, and Cleaved IL-1β. (**E**) Pull-down analysis of the binding between swine Sterp-II-IL-1R1-Flag (sIL-1R1) and mature IL-1β, cleaved IL-1β, with Sterp-II-GFP-Flag as a negative control. (**F**, **G** and **H**) Functional assays of IL-1β. PAMs were treated with 20 ng/mL of the indicated IL-1β proteins for 24 h at 37°C and 5% CO2. The mRNA levels (related to swine GAPDH) of IL-1β (**F**), IL-6 (**G**), and TNF-α (**H**) in the cells were detected by qPCR. (**I** and **J**) The effect of cIL-1β on virus replication and proliferation. (**I**) After treatment of PAMs with sPro-IL-1β, Mature IL-1β, and cleaved IL-1β for 24 hours, PAMs were infected with GFP-SVV at an MOI of 0.1. At 24 hpi, PAMs were observed under a fluorescence inverted microscope (Evos FL Auto2 fluorescence microscope). Bars = 100 μm. (**J**) After treatment of PAMs with sPro-IL-1β, Mature IL-1β, and cleaved IL-1β for 24 hours, PAMs were infected with SVV at an MOI of 0.1. At 24 hpi, the cell culture supernatant was transferred to pre-seeded 96-well plates containing BHK-21 cells for TCID50 determination. A *P* value of less than 0.05 was considered statistically significant. ** for *P*<0.01, *** for *P*<0.001, ns for no significant.

Pro-IL-1β is highly upregulated when the cells were stimulated upon viral infection or cellular stress, mediated NF-κB activation. After the second stimulation by NLRP3 ligands or LPS induced non-canonical inflammasome, endogenous caspase-1 cleaves Pro-domain of hPro-IL-1β to produce active IL-1β. However, the biological activity of swine IL-1β (cIL-1β) after SVV 3C cleavage was not clear. We established an *in vitro* system that separated the incubation products of recombinant sPro-IL-1β and SVV 3C. The cIL-1β and extra SVV 3C were separated by the gel filtration chromatography (Fig 5A). Since the sPro-IL-1β recombinant protein forms an oligomerized state as shown by gel filtration chromatography (Fig 2F), we sought to test whether sPro-IL-1β is oligomerized in the cells. SPro-IL-1β and hPro-IL-1β were transfected into HEK-293T cells, and subsequently native gel analysis showed that intracellular sPro-IL-1β also formed an oligomer while hPro-IL-1β formed a monomer (Fig 5B). Although sPro-IL-1β forms oligomers when expressed in the *E*. *coli*, cIL-1β was present as a monomer, consistent with the mature IL-1β, which we purified from E. coli (Figs 5C, 5D and S5G). Previous observation that sPro-IL-1β lacking residues 1–89 is monomeric (S5E Fig), this suggests that the pro-domain is the key determinant in the formation of sPro-IL-1β oligomers. This also explains why cIL-1β is monomeric after the pro-domain is cleaved by SVV 3C. Mature IL-1β is secreted into the extracellular space, where it binds to receptor IL-1R1, activating immune cells to produce inflammatory cytokines. To explore whether cIL-1β can bind to receptor IL-1R1 and activate the pathway, we expressed Strep-II-IL-1R1-Flag in HEK-293T cells and purified the protein by affinity chromatography column. Subsequently, we conducted a pull-down assay by using recombinant Strep-II-sIL-1R1-Flag protein co-immunoprecipitated with cIL-1β and mature IL-1β, with GFP protein serving as a negative control. The results indicated that both mature IL-1β and cleaved IL-1β can bind to receptor sIL-1R1 (Fig 5E). To further test the biological function of the cIL-1β, PAMs were treated with purified sPro-IL-1β, purified IL-1β, and cIL-1β for 24 h. The mRNA level of IL-1β was determined by qPCR. The results showed that the mRNA level for IL-1β, IL-6, and TNF-α were significantly increased, when treated with cIL-1β similar to cells treated with mature IL-1β (Fig 5F–5H). To further determine the impact of cIL-1β on SVV replication, PAMs were treated with sPro-IL-1β, mature IL-1β, and cIL-1β proteins for 24 h, followed by GFP-SVV infection for 24 h. Fluorescence microscopy analysis of the GFP-SVV spots showed a significant reduction of GFP-SVV spots in the cIL-1β-treated group, as well as the mature IL-1β-treated group but not in the sPro-IL-1β group (Fig 5I), which suggests that cIL-1β suppressed SVV replication. To investigate whether cIL-1β suppresses SVV replication, PAMs were treated in the same way, and cell culture supernatants were collected after 24 h post infection for TCID_50_ determination. The results showed that cIL-1β treatment significantly inhibited the viral titers, consistent with mature IL-1β, indicating that cIL-1β significantly inhibits SVV replication (Fig 5J). Collectively, SVV 3C cleavage generates biologically active cIL-1β, which possesses the capacity to inhibit viral replication.

## Discussion

The main function of IL-1 family cytokines is to regulate proinflammatory responses upon stimulation by pathogen-associated molecular patterns (PAMPs) or damage-associated molecular patterns (DAMPs) [24–26]. Upon activation during viral infection, the virus coordinates the inflammatory response by secreting proinflammatory cytokines, particularly IL-1β. IL-1β is initially produced as an inactive precursor (Pro-IL-1β) and is converted into its active form by a suite of proteases, such as caspase-1, neutrophil-derived proteases, and microbial-derived proteases [27]. Our results reveal that the pro-IL-1β serves as an inflammatory sensor for SVV 3C protease, thus initiate inflammation activation. SVV 3C selectively cleaves sPro-IL-1β without affecting hPro-IL-1β and does not cleave Pro-IL-18 or Pro-IL-33, indicating a substrate-specific interaction. While 3C proteases are a common feature among *Picornaviridae* family viruses, this study shows that other 3C proteases in this family do not cleave Pro-IL-1β. In the context of SVV infection, the activation of the inflammasome and the direct cleavage of Pro-IL-1β by SVV 3C occur at the same time. However, when the inflammasome is blocked by the pan-caspase inhibitor, SVV 3C continues to activate IL-1β through direct cleavage, serving as an alternative pathway for eliciting the inflammatory response (Fig 3C and 3D). Extending this inquiry to other viral 3C-like proteases yielded a surprising result: none were capable of cleaving Pro-IL-1β. This specificity suggests that SVV 3C is currently the only viral protease capable of cleaving Pro-IL-1β, which explains why SVV infection causes a stronger inflammatory response [28,29].

The cleavage sites of SpeB have been identified at F105 [12] and at H115 [11], which differ from the caspase-1 cleavage site at D116 in hPro-IL-1β. Utilizing mass spectrometry and site-directed mutagenesis, this study reveals that SVV 3C cleaves sPro-IL-1β by recognizing the "LQ" motif between K123 and L124. This cleavage occurs 9 residues downstream of D114, the cleavage site for swine caspase-1. Interestingly, the cIL-1β produced by SVV 3C, although differs from the mature IL-1β generated by caspase-1, remains directly binds to swine IL-1R1 and can elicit proinflammatory responses in macrophages, which suggests the cIL-1β is biologically active.

Recent studies have elucidated the structural basis of caspase-4-pro-IL-18 complex, offering insights into how substrates are recognized, cleaved and released [8,9]. In our effort to visualize the complex of SVV 3C-sPro-IL-1β using cryo-electron microscopy (cryo-EM), we encountered heterogeneous conformations, which posed a challenge for resolving high resolution structures. Nevertheless, we constructed a model of the SVV 3C-sPro-IL-1β complex that highlights the essential role of the sIL-1β pro-domain in recognition and cleavage by 3C. By generating a series of Pro-IL-1β truncations and mutants, we determined that residues 109–114 of the pro-domain plays a significant role, akin to the recognition of the IL-18 precursor by caspase-4.

When Pro-IL-1β is cleaved by caspase-1/4/5, it not only generates mature IL-1β (p17) but also produces a 27 kDa fragment (p27). Under physiological condition, mouse caspase-11 can only process Pro-IL-1β to produce p27 but not p17 [22]. In our study, we discovered that SVV 3C can cleave both sPro-IL-1β and hPro-IL-1β to produce p27. However, whether p27 promotes or impedes IL-1β signaling remains to be further investigated.

IL-1β plays a critical role in host-pathogen interactions, especially in pathological injury. In bacterial infections, proteases from GAS and *Pseudomonas aeruginosa* can directly cleave Pro-IL-1β, providing new insights into how bacterial infections cause the host high inflammation and pus formation [11–13]. Normally, the canonical inflammasome pathway has been considered the mechanism for activating IL-1β upon viral infection. However, our findings reveal that SVV 3C protease can also directly cleave Pro-IL-1β to produce IL-1β, especially when the canonical inflammasome pathway is inhibited.

**Fig 6:**
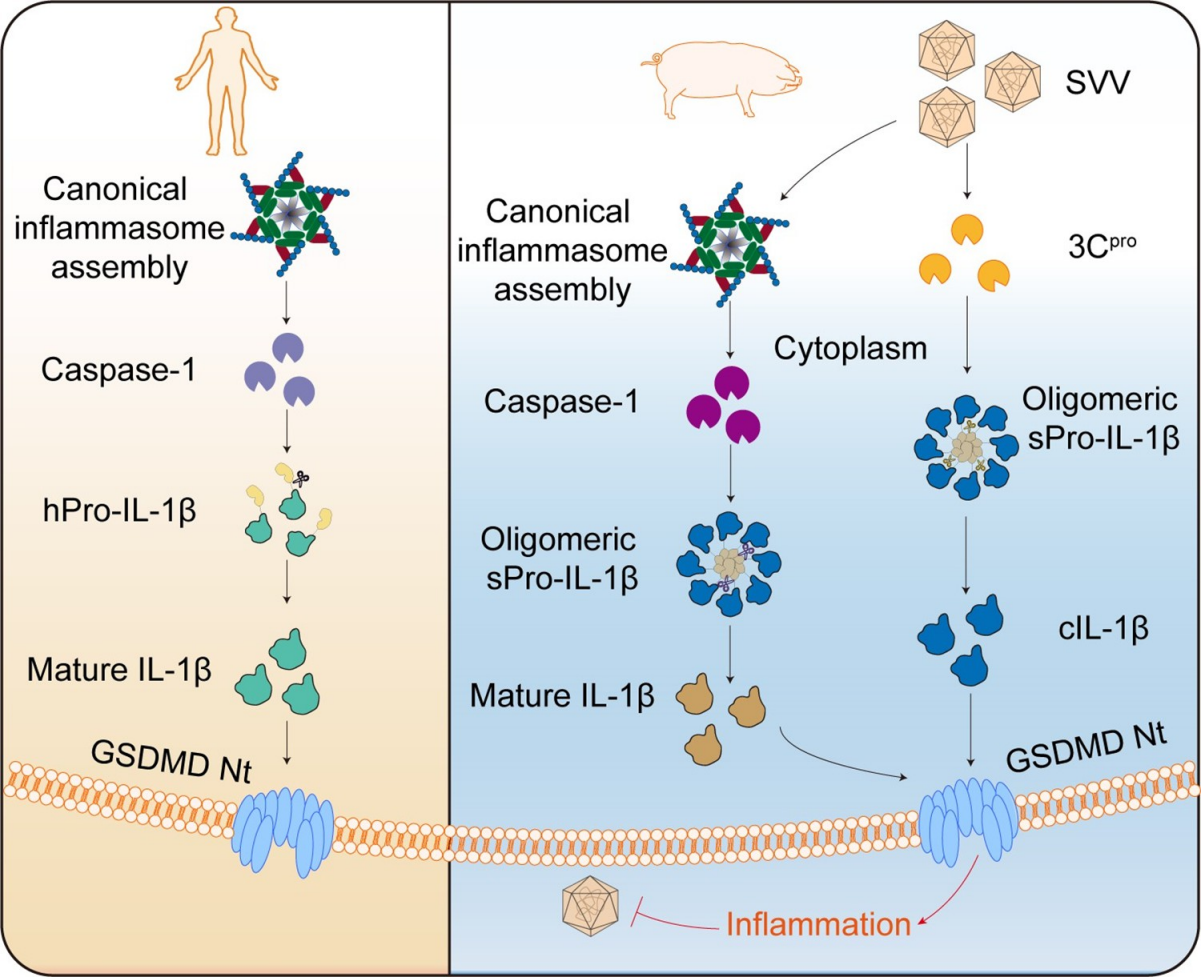
Schematic model of the oligomeric sPro-IL-1β were cleaved by SVV 3C protease into the cIL-1β. Swine Pro-IL-1β is an inflammatory sensor that directly detects SVV 3C protease through an independent pathway operating in parallel with host inflammasomes

In conclusion, this study demonstrates that swine Pro-IL-1β is an inflammatory sensor that directly detects SVV 3C protease through an independent pathway operating in parallel with host inflammasomes (Fig 6). Despite the high sequence homology of Pro-IL-1β between swine and human, the distinct structure of pro-domain of swine Pro-IL-1β resulting in recognition of substrates by SVV 3C. However, further structural studies are needed to show how swine Pro-IL-1β oligomerizes and how SVV 3C protease recognizes the substrate. The unique mechanism of swine IL-1β maturation elucidated in this study provides valuable insights into the processing and activation of IL-1β, advancing our understanding of innate immunity.

## Materials and methods

### Cell lines

HEK-293T cells, BHK-21 cells and PAM-Tang cells were cultured in Dulbecco’s Modified Eagle Medium (DMEM) (MACGENE, #CM10013) containing 10% fetal bovine serum (FBS) (PlantChemMed, #PC-00001), with 100 U/ml penicillin and 100 μg/ml streptomycin (Solarbio, #P1400) at 37°C with 5% CO2. Porcine Alveolar Macrophages (PAMs) were isolated from 4-week-old specific pathogen-free piglets and cultured in RPMI 1640 medium containing 10% fetal bovine serum (with 100 U/ml penicillin and 100 μg/ml streptomycin). HEK-293T cell lines stably expressing swine Pro-IL-1β (sPro-IL-1β-293T) were constructed using Lentiviral over-expression system. PAM-Tang cells were a gift from Professor Yandong Tang from Harbin Veterinary Research Institute (China). Cell images were analyzed using Falcon S300, Intelligent cell imaging and analysis system (Alicelligent Technologies).

### Ethics statement

Porcine Alveolar Macrophages (PAMs) were isolated according to the protocols, which were approved by the Committee on the Ethics of Animal Experiments of the Harbin Veterinary Research Institute (HVRI) of the Chinese Academy of Agricultural Sciences (CAAS) and the Animal Ethics Committee of Heilongjiang Province, China. The license number associated with this research protocol was 231017-01-GR.

### Virus and infection

The Seneca Valley virus (SVV) and Encephalomyocarditis virus (EMCV) were stored in our laboratory. PAMs, PAM-Tang cells and sPro-IL-1β-293T cells were infected with SVV and EMCV for different time points.

### Plasmids and transfection

DNAs encoding swine IL-1β, swine IL-18, swine IL-33, human IL-1β, NSP5 of PDCoV and PEDV, pS273R of ASFV, NSP2 and NSP4 of PRRSV were ligated in the pcDNA3.1(+) vectors. For stable expression, DNAs encoding swine IL-1β were ligated in PNL-GFP vector. DNAs encoding 3C protease of SVV, EMCV, EV71, HAV, PV-1, PV-3 were stored by our laboratory. DNA encoding NSP5 of SARS-Cov-2 were synthesized by Beijing Tsingke.

For screening cleavage site and interaction site, DNAs encoding swine IL-1β-C122A, K123A, L124A, Q125A, D126A, KL124AA, LQ125AA, KLQ125AAA, KLQD126AAAA, F95A, FEE99AAA, I102A, IL104AA, C107A, C113A, DDF111AAA, DDFVC113AAAAA, D114A, Δ41, Δ83, Δ89, Δ90–115 were ligated in the pcDNA3.1(+) vectors.

For recombinant expression Escherichia coli, DNAs encoding the swine Pro-IL-1β, mature-IL-1β, and mutant IL-1β (L124A, LQ125AA, KLQD126AAAA), human Pro-IL-1β, SVV 3C WT and catalytically deficient domain of 3C (H48A, D84A, C160A and D84A/C160A) were cloned into 6×His-SUMO-pET28a vector.

According to the manufacturer’s instructions, transient transfection of the above plasmids was performed using Lipofectamine 2000 (Thermo Fisher Scientific, #11668500). To establish stable expression cell lines, the lentiviral plasmid containing the desired gene was co-transfected with Packaging and VSVG plasmids at a 1:1:1 ratio into HEK-293T cells. After 48 h post-transfection, the supernatants were collected and used to infect indicated cells for another 48h in the presence of polybrene, followed by identification assays.

### Reagents and antibodies

LPS (HY-D1056), Nigericin (HY-127019) and Pan-caspase inhibitor Z-VAD (HY-16658) were obtained from MedChemExpress. Swine IL-1β ELISA KIT (SEKP-0001) was purchased from Solarbio.

Antibodies against HA tag (51064-2-AP), Flag tag (20543-1-AP), CoraLite488-conjugated Goat Anti-Rabbit IgG (SA00013-2), CoraLite594-conjugated Goat Anti-Rabbit IgG (SA00013-4), CoraLite488-conjugated Goat Anti-Mouse IgG (SA00013-1), CoraLite594-conjugated Goat Anti-Mouse IgG (SA00013-3) were purchased from Proteintech. Antibodies against β-actin (sc-47778) and human IL-1β (sc-32294) were obtained from Santa Cruz. Antibodies against SVV 3C were obtained from SinoBiological. Antibodies against IL-1β (WLH3903) and caspase-1 (WL02996a) were purchased from WanleiBio. Antibody against Flag tag (F1804) was purchased from Sigma. Antibodies against HA tag (3724S) was purchased from Cell Signaling Technology. Antibodies against swine IL-1β were produced by our laboratory.

### Recombinant protein expression and purification

The cDNA encoding the full-length Pro-IL-1β, mutant Pro-IL-1β (L124A, LQ125AA, KLQD126AAAA, Δ83, Δ89, Δ90–115), mature IL-1β of swine and human Pro-IL-1β was cloned into the pET-28a expression vector, and its C-terminal was fused with the SUMO-tag. The target protein is successfully expressed in soluble form after induction by transforming the Escherichia coli BL21 (DE3) (Tsingke Biotech Co., Ltd.). Specifically, positive BL21 (DE3) was cultured in LB medium 30 μg/ml kanamycin until OD600 value reached 1.0 and 0.4 mM β-D-1-thiogalactopyranoside (IPTG) was added, then the target protein was expressed at 16°C for about 20 h. The cells were resuspended and disrupted by sonication in lysis buffer (50mM Tris-HCl pH 8.0, 300mM NaCl) and centrifuged at 4°C for 10 mins at 12,000 g to separate the supernatants. The target protein in the supernatant was purified with Ni-NTA affinity chromatography (Qiagen), then washed beads with washing buffer (20mM Tris-HCl pH 7.5, 500mM NaCl, 25mM Imidazole) and finally the protein was collected with elution buffer (20mM Tris-HCl pH 7.5, 150mM NaCl, 250mM Imidazole). The SUMO-tag was removed by adding the SUMO protease digestion overnight at 4°C. Finally, the protein was further purified by gel filtration chromatography on Hiload 16/600 Superdex 200pg (GE Healthcare Life Sciences) with running buffer (20 mM Tris-HCl pH 7.5, 150 mM NaCl). All collected proteins need to be concentrated after adding 5 mM DTT, immediately frozen in liquid nitrogen stored at -80°C till further use. SVV 3C and its mutant proteins (H48A, D84A, C160A and H48A/C160A) are stored in our laboratory.

Recombinant expression and purification of the catalytically active swine caspase-1 (sCaspase-1) p20/p10 complex were performed using a previously reported refolding method [30]. In brief, the untagged P20 was cloned into the pET-28a vector and P10 into the pET-21a vector, and each was expressed in Escherichia coli BL21 (DE3) separately. Expression was induced with IPTG at 37°C for 4 hours. Equal molar amounts of each subunit were mixed to a total volume of 5 mL and rapidly diluted into 250 mL of refolding buffer (100 mM HEPES (pH 8.0), 100 mM NaCl, 100 mM sodium malonate, 20% sucrose, 0.5 M NDSB-201, and 10 mM DTT). The refolded proteins were concentrated and dialyzed overnight in a buffer (30 mM sodium acetate pH 5.9, 10% (v/v) glycerol), and then exchanged into a buffer (72 mM sodium acetate pH 5.9, 400 mM sodium malonate, and 10% (v/v) glycerol) by RESOURCE S cation exchange chromatography (GE Healthcare Life Sciences). The P20/P10 were further purified by Hiload 16/600 Superdex 200pg gel-filtration chromatography (GE Healthcare Life Sciences).

To recombinantly express swine IL-1R1 and GFP proteins in mammalian cells, Strep-II-IL-1R1-Flag and Strep-II-GFP-Flag were cloned into the pcDNA3.1 vector. Successfully constructed plasmids were transfected into 293T cells, which were then cultured at 37°C in a 5% CO_2_ incubator for 48 hours. After cell lysis with NP-40, the lysates were centrifuged at 5000 rpm for 10 minutes, and the supernatants were collected for purification using Strep-Tactin XT (IBA Lifesciences, #2-5032-001). The target proteins were eluted with a buffer containing 100 mM Tris-HCl (pH 8.0), 150 mM NaCl, 1 mM EDTA, and 50 mM biotin. The eluted target proteins were collected, characterized, and stored at -80°C.

### SDS-PAGE, Blue Native PAGE and Western Blotting

The protein samples were prepared and separated using SDS-PAGE with 12.5% gels, following the experimental protocol provided by Shanghai Epizyme Biomedical Technology (Shanghai, China). The separated samples were used for Coomassie Brilliant blue and Western blotting, respectively.

For Coomassie Brilliant Blue staining, the protein samples were incubated overnight at room temperature in a staining solution on a shaker. Subsequently, the stained gels were destained on a shaker in a destaining solution for 1 hour before observation.

For Blue Native PAGE, the protein samples were prepared and separated using Precast-Gel (Solarbio, #PG42015-N).

For Western blotting, the separated protein samples were transferred onto PVDF membranes (0000223060, Immobilon). After blocking with 5% skim milk in PBST (0.01 M phosphate-buffered saline (PBS), pH 7.2, 0.05% Tween 20) at room temperature for 2 h, the membranes were incubated overnight with a monoclonal antibody at 4°C. Following washing, the membranes were incubated with Anti-mouse/rabbit IgG HRP-Linked Antibody (7076/7074, Cell Signaling Technology) at 37°C for 1 hour, respectively. The targeted proteins on the membranes were then detected using a chemiluminescence detection kit (170–5061, BIO-RAD).

### RNA isolation and quantitative real-time PCR (RT-qPCR)

RNA was extracted from whole-cell lysates using the RNA simple Total RNA Kit (TIANGEN, #DP419) and subsequently reverse transcribed to cDNA using the HiScript II Q RT SuperMix for qPCR (+gDNA wiper) Kit (Vazyme, #R223-01). Quantitative PCR (qPCR) was conducted using the ChamQ SYBR qPCR Master Mix (Vazyme, #Q712-02). Threshold cycle numbers were normalized using triplicate samples amplified with primers specific to GAPDH.

### Cytotoxicity, IL-1β detection

Cell death was measured by LDH assay using CytoTox 96 Non-Radioactive Cytotoxicity Assay (Promega, #G1780). In a 96-well plate, 50 μL of cell culture supernatant was mixed with 50 μL of LDH assay buffer, followed by an incubation at 37°C for 30 minutes. The reaction was stopped by adding the stop solution, and the absorbance was measured using a microplate reader (Bio-Rad).

To measure the IL-1β release, Cell culture supernatants were collected and measured by using Porcine IL-1β ELISA KIT (Solarbio, #SEKP-0001).

### IL-1β bioactivity assay

To measure the biological activity of IL-1β generated by cleavage, the products of cleavage reaction are collected and the proteins are separated using Superdex 200 Increase 10/300 GL (GE Healthcare Life Sciences). Swine Alveolar Macrophages (PAMs) are stimulated with 20 ng/mL of specified IL-1β at 37°C and 5% CO2 for 24 h. Subsequently, cells are collected for quantitative real-time PCR analysis.

### Binding studies by gel filtration chromatography

For the binding studies, the sPro-IL-1β protein samples (at ~300 μM) were mixed with equal volume of SVV 3C DM protein (at ~150 μM or 300 μM) on ice for 2 h. The mixtures of samples were injected over Superdex200 (10/300 GL) column (GE Healthcare Life Sciences) eluted with running buffer (20 mM Tris-HCl pH 7.5, 150 mM NaCl). The elution fractions were analyzed by SDS–PAGE.

### Recombined Swine NLRP3 inflammasome activation

HEK293T cells were seeded into a 24-well plate (5×105 cells per well) and incubated overnight. pcDNA3.1-NLRP3-HA (50 ng), pcNDA3.1-ASC (5 ng), pCAGGS-Caspase-1 (3 ng), pcNDA3.1-Pro-IL-1β (200 ng) were co-transfected with 200 ng SVV 3C and EMCV 3C. For positive control, HEK293T cells were transfected for 12 h, exposed to LPS (100 ng/mL) for 8 h, and then treated with nigericin for 4 h. Cells were collected and subjected to Western Blotting at 24 h after transfection.

### *In vitro* cleavage assay and mass spectrometry

Cleavage of swine Pro-IL-1β, human Pro-IL-1β, mutant Pro-IL-1β (L124A, LQ125AA, KLQD126AAAA) and deletion Pro-IL-1β (Δ83, Δ89 and Δ90–115 sPro-IL-1β) by purified SVV 3C WT were performed in a 25 μL reaction containing 50 mM HEPES (pH 7.5), 3 mM EDTA, 150mM NaCl, 0.005% (vol/vol) Tween-20 and 10 mM DTT at 37°C for 2 h. Substrate protein(25 μM) were reacted with SVV 3C at multiple concentrations. The cleavage reaction was terminated by adding 5×SDS Loading buffer, then boiled for 10 minutes in preparation for SDS-PAGE. The major cleavage band, approximately 17 kDa, was excised from the SDS-PAGE gel and subjected to trypsin protease digestion for mass spectrometry analysis. The mass spectrometry results were analyzed by Matrix Science.

### Co-immunoprecipitation

After washing cells with 1×PBS, they were lysed in NP-40 buffer at 4°C for 30 minutes. The supernatant was collected after centrifugation. One-fifth was used as input, the rest incubated with ProteinA/G and IgG antibodies at 4°C for 4–6 h for purification. After centrifugation at 1000g for 5 minutes at 4°C, the supernatant was enriched overnight at 4°C with Flag antibody (Sigma, # F1804) and ProteinA/G (Santa Cruz, sc-2003). The enriched samples were then washed, and 80 μL fresh NP-40 buffer plus 5×SDS-loading buffer were added for Western Blotting analysis.

### Confocal microscopy

After co-transfection of Pro-IL-1β and 3C into 293T cells for 24 h, 4% paraformaldehyde solutions were used to fix the monolayer of cells at room temperature for 15 minutes. Subsequently, cells were permeabilized using 0.1% Triton X-100. To investigate the co-localization of Pro-IL-1β with SVV 3C, staining was performed using IL-1β antibody (WLH3903, WanleiBio) and Flag antibody. Confocal microscopy (Nikon A1, Japan) was utilized for image acquisition and analysis.

### Pull-down assays

Purified mature IL-1β and cleaved IL-1β were incubated with purified Strep-II-IL-1R1-Flag and Strep-II-GFP-Flag proteins at 4°C for 6 hours. Subsequently, Flag monoclonal antibody (Sigma, # F1804) was added and the mixture was incubated overnight. The next day, Protein A/G was added and incubated for 6 hours at 4°C to enrich the samples. The enriched samples were then washed, and 80 μL of fresh NP-40 buffer plus 5× SDS-loading buffer were added for Western Blotting analysis.

### Statistical analysis

The duplicate experimental samples were subjected to t-test analysis to determine the means ± standard deviation (SD) using GraphPad Prism 9.5 software. Significance levels were denoted as follows: * for *P<*0.05, ** for *P<*0.01, and *** for *P<*0.001. ns for no significance.

## Supporting information

S1 Fig Protein purification of full-length SVV 3C, swine Pro-IL-1β, human Pro-IL-1β and swine p20/p10. (A to D) SDS-PAGE analysis of SVV 3C (A), swine Pro-IL-1β (B), human Pro-IL-1β (C) and swine caspase-1 (sCaspase-1) p20/p10 (D) purified by gel filtration chromatography (left), while SVV 3C (A), human Pro-IL-1β (C) and swine caspase-1 (sCaspase-1) p20/p10 (D) were further purified by ion exchange chromatography (middle).
(TIF)

S2 Fig hPro-IL-1β, sPro-IL-18 and sPro-IL-33 are not cleaved by SVV 3C to produce mature cytokines. (A)Western blotting analysis of in vitro cleavage of sPro-IL-1β in reaction buffer containing 25 μM sPro-IL-1β recombinant protein with purified recombinant protein SVV 3C for 2 h at 37°C. (B) HEK-293T cells were transfected with a plasmid encoding sPro-IL-1β, together with a plasmid encoding EMCV 3C. (C) HEK-293T cells were transfected with plasmids encoding hPro-IL-1β and SVV 3C. (D) SDS-PAGE analysis of in vitro cleavage of hPro-IL-1β in a reaction buffer containing 25 μM hPro-IL-1β recombinant protein with different dose of purified recombinant protein SVV 3C (0.25, 1, 2, 4, 6, 8, 12 μM) for 2 h at 37°C. (E) HEK-293T cells were transfected with plasmids encoding SVV 3C and sPro-IL-18. (F) HEK-293T cells were transfected with plasmids encoding SVV 3C and sPro-IL-33. (A, B, C, E and F) The cleavage of Pro-IL-1β were detected by Western Blotting using anti-sIL-1β, anti-hIL-1β, anti-Flag and anti-β-actin antibodies.
(TIF)

S3 Fig sPro-IL-1β cannot be cleaved by other viral proteases. (A and B) HEK-293T cells were transfected with a plasmid encoding hPro-IL-1β, together with a plasmid encoding EV71 3C (A), HAV 3C (A), PV-1 3C (A), PV-3 3C (A) and SARS-CoV2-NSP5 (B). (C) HEK-293T cells were transfected with a plasmid encoding sPro-IL-1β, together with plasmids encoding PDCoV-NSP5, PEDV-NSP5, ASFV-pS273R, PRRSV-NSP4 and PRRSV-NSP2. (D) HEK-293T cell lines stably expressing swine Pro-IL-1β (sIL-1β-293T) were constructed using Lentiviral over-expression system. The success of the construction was verified by Western blotting. (**E**) SDS-PAGE analysis of *in vitro* cleavage of 25 μM sPro-IL-1β recombinant protein or its mutants (L124A, LQ125AA and KLQD126AAAA) recombinant protein with purified recombinant protein sCaspase-1 p20/p10 for 2 h at 37°C.
(TIF)

S4 Fig Interaction between sPro-IL-1β and SVV 3C *in vitro*. (A and B) Western blotting (A) and Blue Native PAGE (B) analysis of sPro-IL-1β binding with SVV 3C double mutants (3C-DM) by gel filtration chromatography. Elution profiles of sPro-IL-1β is in black. Elution profiles of mixtures of sPro-IL-1β and 3C-DM at molar ratios 2:1 and 2:2 are in blue and red, respectively.
(TIF)

S5 Fig Protein purification of swine mutant Pro-IL-1β and Mature IL-1β. SDS-PAGE analysis of sPro-IL-1βL^124^A (A), sPro-IL-1βLQ^125^AA (B), sPro-IL-1βKLQD^126^AAAA (C), Δ83 sPro-IL-1β (D), Δ89 sPro-IL-1β (E), Δ90–115 sPro-IL-1β (F) and Mature IL-1β (G) purified by gel filtration chromatography.
(TIF)

S6 Fig Sequence and structural analysis of sPro-IL-1β recognition by SVV 3C protease. (A) Sequence alignment of the structural domains of sPro-IL-1β and hPro-IL-1β. Identical and similar amino acids are highlighted in red or white boxes, respectively. The alignment was performed using the ClustalOmega online tool. (B) The overall structure of SVV 3C is shown in surface representation with the 109-DNEAYV-114 motif of hPro-IL-1β shown in stick representation. Positively charged pocket (H48, R150, K157, H178 of SVV 3C) was marked in hotpink on surface model. (C and D) Analysis of the surface electrostatics of SVV 3C. The 109-DDFVCD-114 motif of sPro-IL-1β inserts into the positively charged pocket of SVV 3C (C), while the corresponding segment 109-DNEAYV-114 motif of hPro-IL-1β is distanced from the positively charged pocket of SVV 3C (D). Positively charged surface is colored blue and negatively charged surface red. The 109-DDFVCD-114 motif of sPro-IL-1β and 109-DNEAYV-114 motif of hPro-IL-1β are shown by the stick models.
(TIF)
