# Supplementary figures and images for: Species-specific IL-1β is an inflammatory sensor of Seneca Valley Virus 3C Protease
Source: PLoS Pathog. 2024 Jul 22;20(7):e1012398. doi: 10.1371/journal.ppat.1012398 (PMC11293702; doi:10.1371/journal.ppat.1012398)

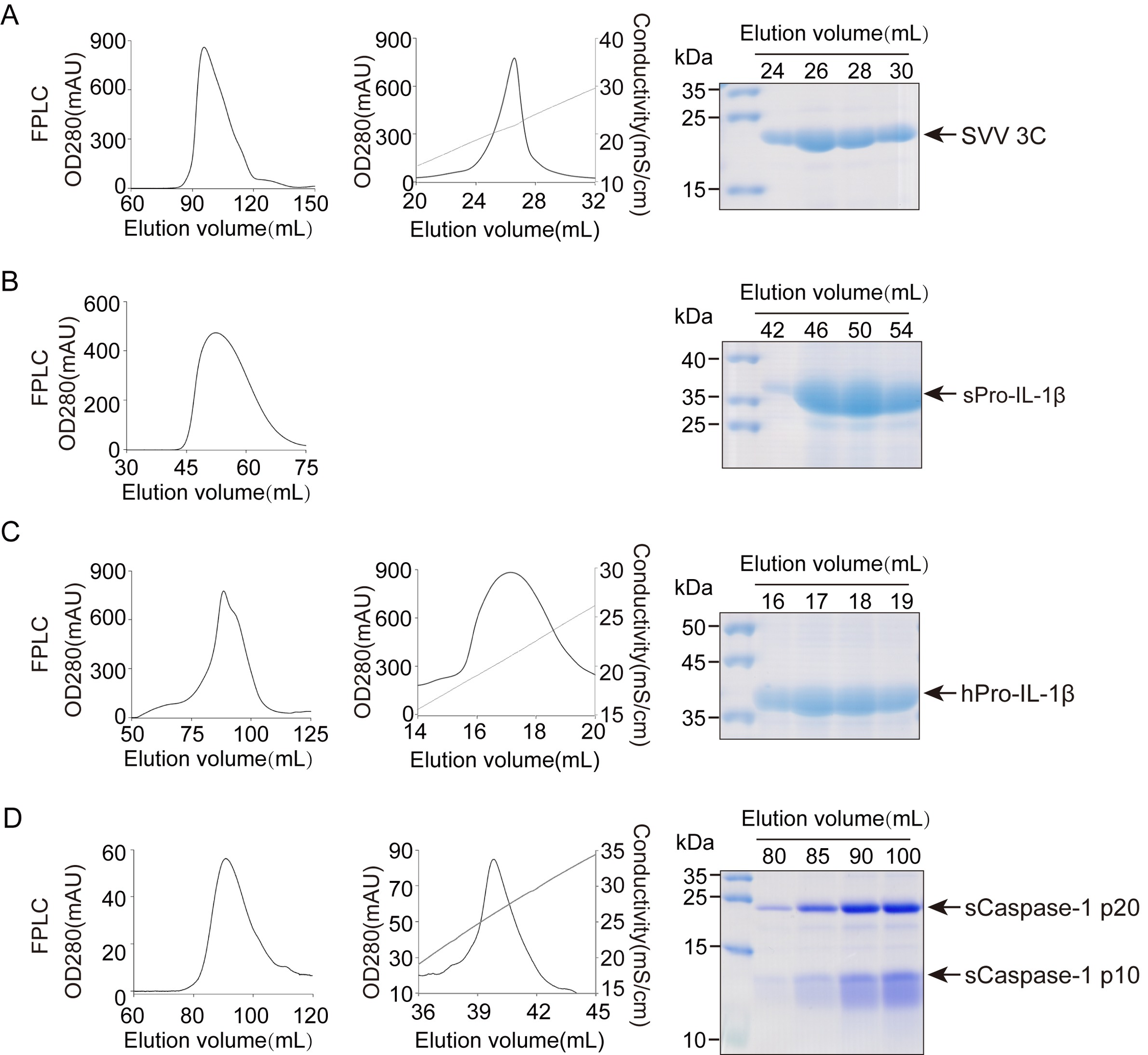

Supplement: S1 Fig — (A to D) SDS-PAGE analysis of SVV 3C (A), swine Pro-IL-1β (B), human Pro-IL-1β (C) and swine caspase-1 (sCaspase-1) p20/p10 (D) purified by gel filtration chromatography (left), while SVV 3C (A), human Pro-IL-1β (C) and swine caspase-1 (sCaspase-1) p20/p10 (D) were further purified by ion exchange chromatography (middle). (TIF) [file ppat.1012398.s001.tif]

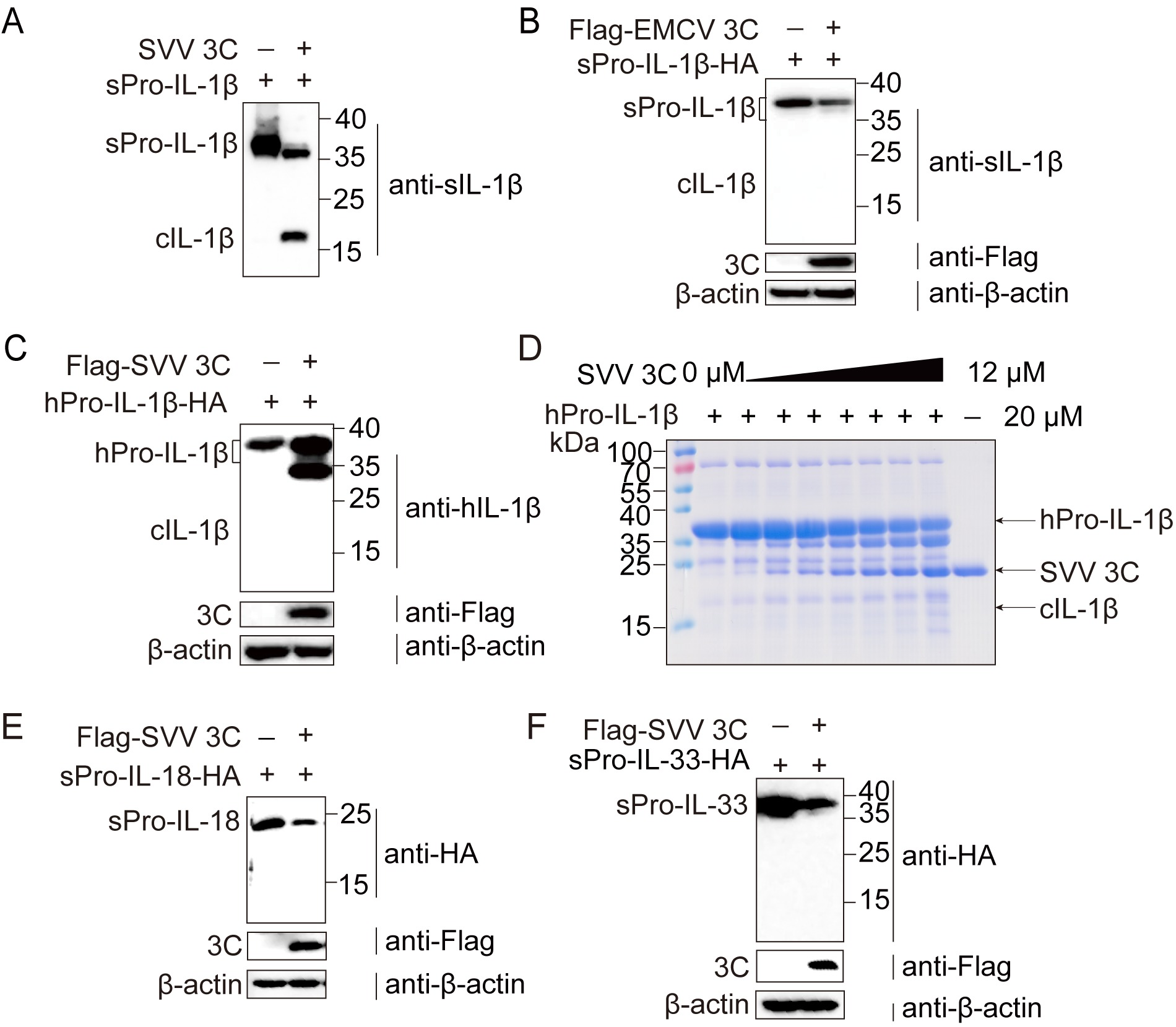

Supplement: S2 Fig — (A)Western blotting analysis of in vitro cleavage of sPro-IL-1β in reaction buffer containing 25 μM sPro-IL-1β recombinant protein with purified recombinant protein SVV 3C for 2 h at 37°C. (B) HEK-293T cells were transfected with a plasmid encoding sPro-IL-1β, together with a plasmid encoding EMCV 3C. (C) HEK-293T cells were transfected with plasmids encoding hPro-IL-1β and SVV 3C. (D) SDS-PAGE analysis of in vitro cleavage of hPro-IL-1β in a reaction buffer containing 25 μM hPro-IL-1β recombinant protein with different dose of purified recombinant protein SVV 3C (0.25, 1, 2, 4, 6, 8, 12 μM) for 2 h at 37°C. (E) HEK-293T cells were transfected with plasmids encoding SVV 3C and sPro-IL-18. (F) HEK-293T cells were transfected with plasmids encoding SVV 3C and sPro-IL-33. (A, B, C, E and F) The cleavage of Pro-IL-1β were detected by Western Blotting using anti-sIL-1β, anti-hIL-1β, anti-Flag and anti-β-actin antibodies. (TIF) [file ppat.1012398.s002.tif]

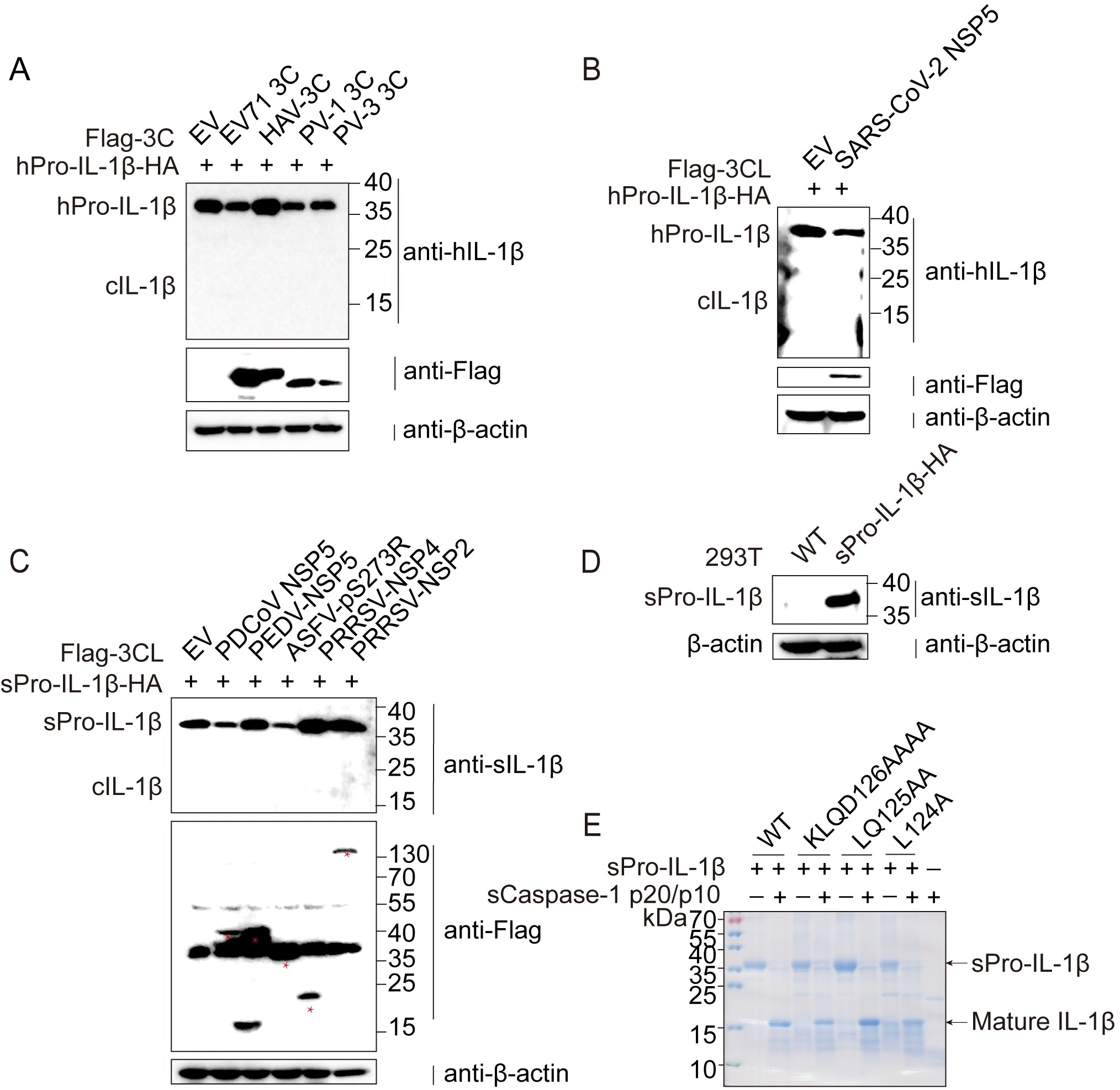

Supplement: S3 Fig — (A and B) HEK-293T cells were transfected with a plasmid encoding hPro-IL-1β, together with a plasmid encoding EV71 3C (A), HAV 3C (A), PV-1 3C (A), PV-3 3C (A) and SARS-CoV2-NSP5 (B). (C) HEK-293T cells were transfected with a plasmid encoding sPro-IL-1β, together with plasmids encoding PDCoV-NSP5, PEDV-NSP5, ASFV-pS273R, PRRSV-NSP4 and PRRSV-NSP2. (D) HEK-293T cell lines stably expressing swine Pro-IL-1β (sIL-1β-293T) were constructed using Lentiviral over-expression system. The success of the construction was verified by Western blotting. (E) SDS-PAGE analysis of in vitro cleavage of 25 μM sPro-IL-1β recombinant protein or its mutants (L124A, LQ125AA and KLQD126AAAA) recombinant protein with purified recombinant protein sCaspase-1 p20/p10 for 2 h at 37°C. (TIF) [file ppat.1012398.s003.tif]

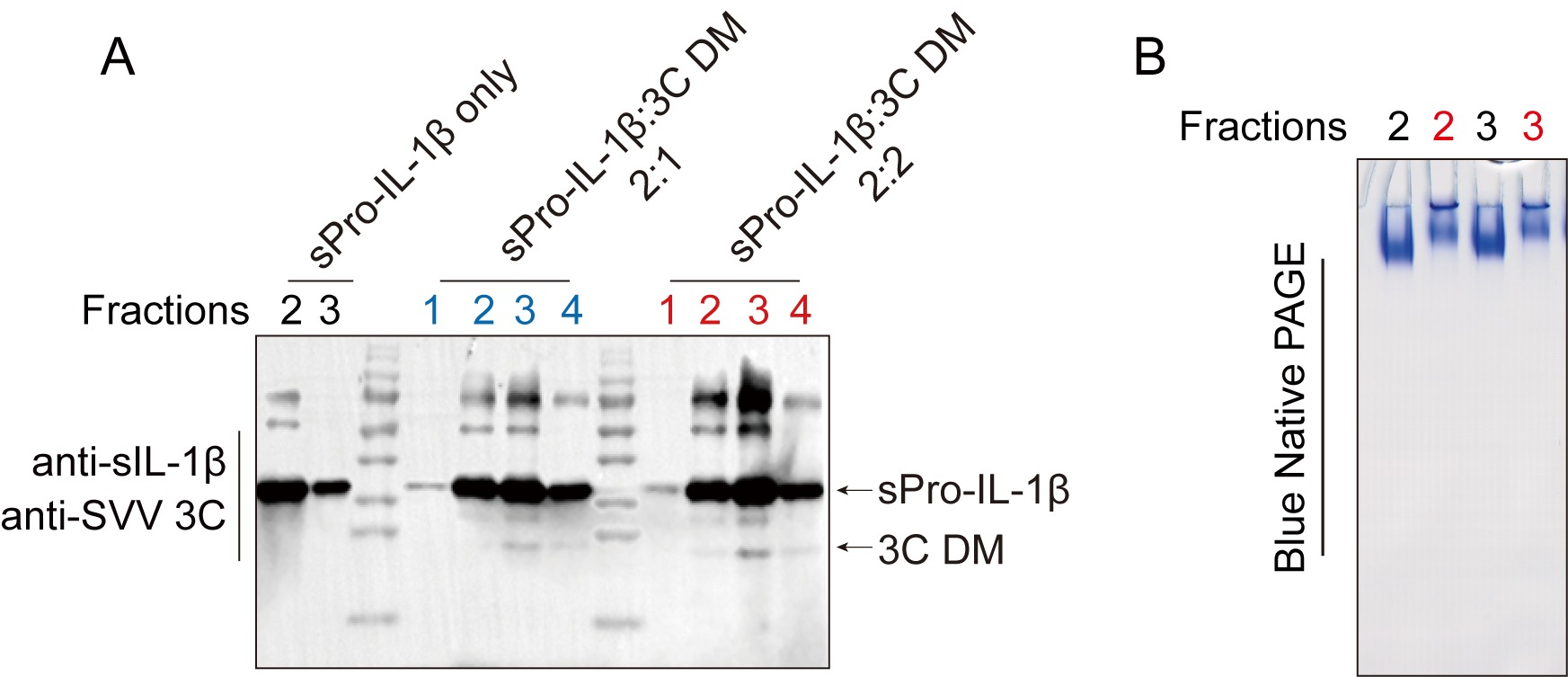

Supplement: S4 Fig — (A and B) Western blotting (A) and Blue Native PAGE (B) analysis of sPro-IL-1β binding with SVV 3C double mutants (3C-DM) by gel filtration chromatography. Elution profiles of sPro-IL-1β is in black. Elution profiles of mixtures of sPro-IL-1β and 3C-DM at molar ratios 2:1 and 2:2 are in blue and red, respectively. (TIF) [file ppat.1012398.s004.tif]

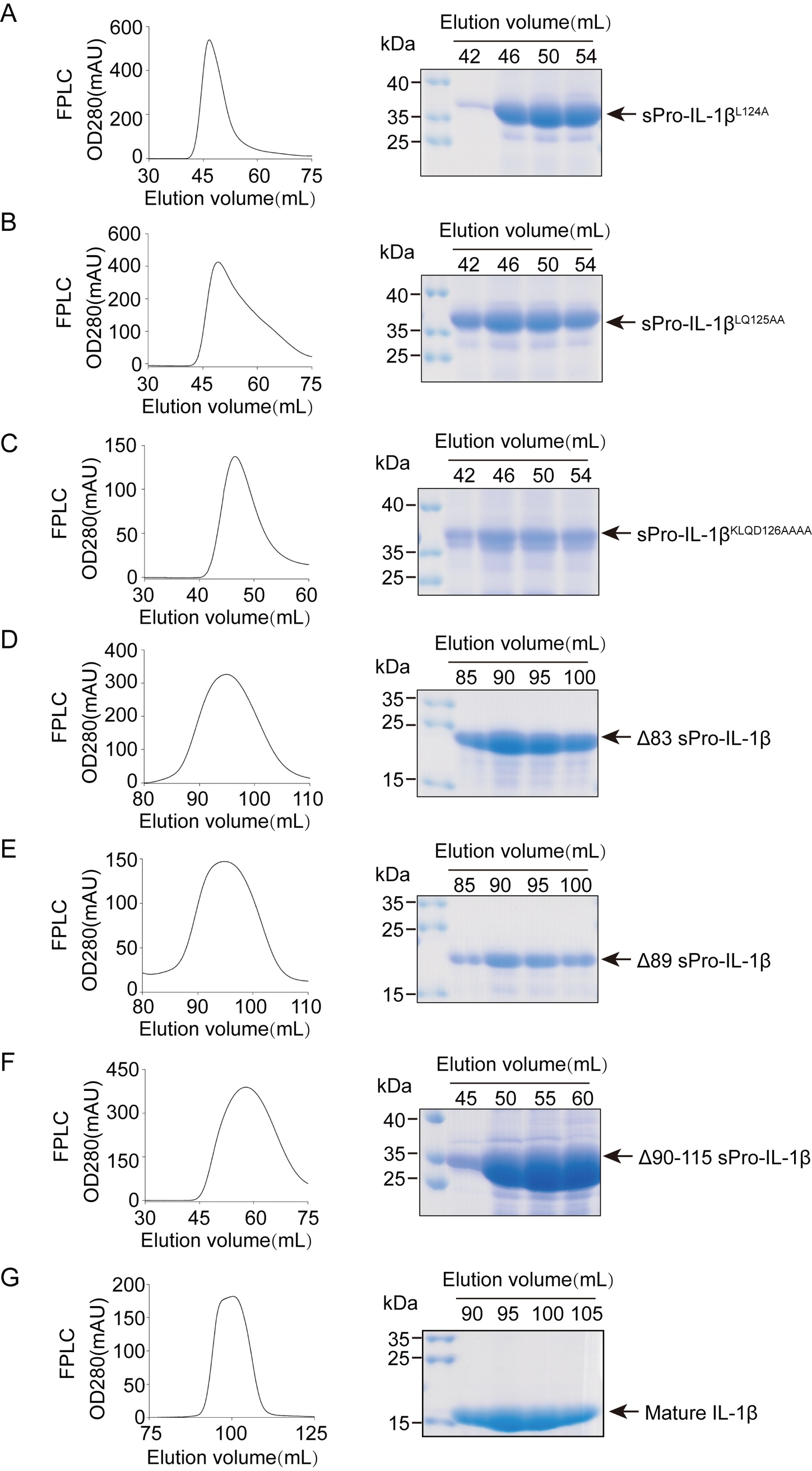

Supplement: S5 Fig — SDS-PAGE analysis of sPro-IL-1βL124A (A), sPro-IL-1βLQ125AA (B), sPro-IL-1βKLQD126AAAA (C), Δ83 sPro-IL-1β (D), Δ89 sPro-IL-1β (E), Δ90–115 sPro-IL-1β (F) and Mature IL-1β (G) purified by gel filtration chromatography. (TIF) [file ppat.1012398.s005.tif]

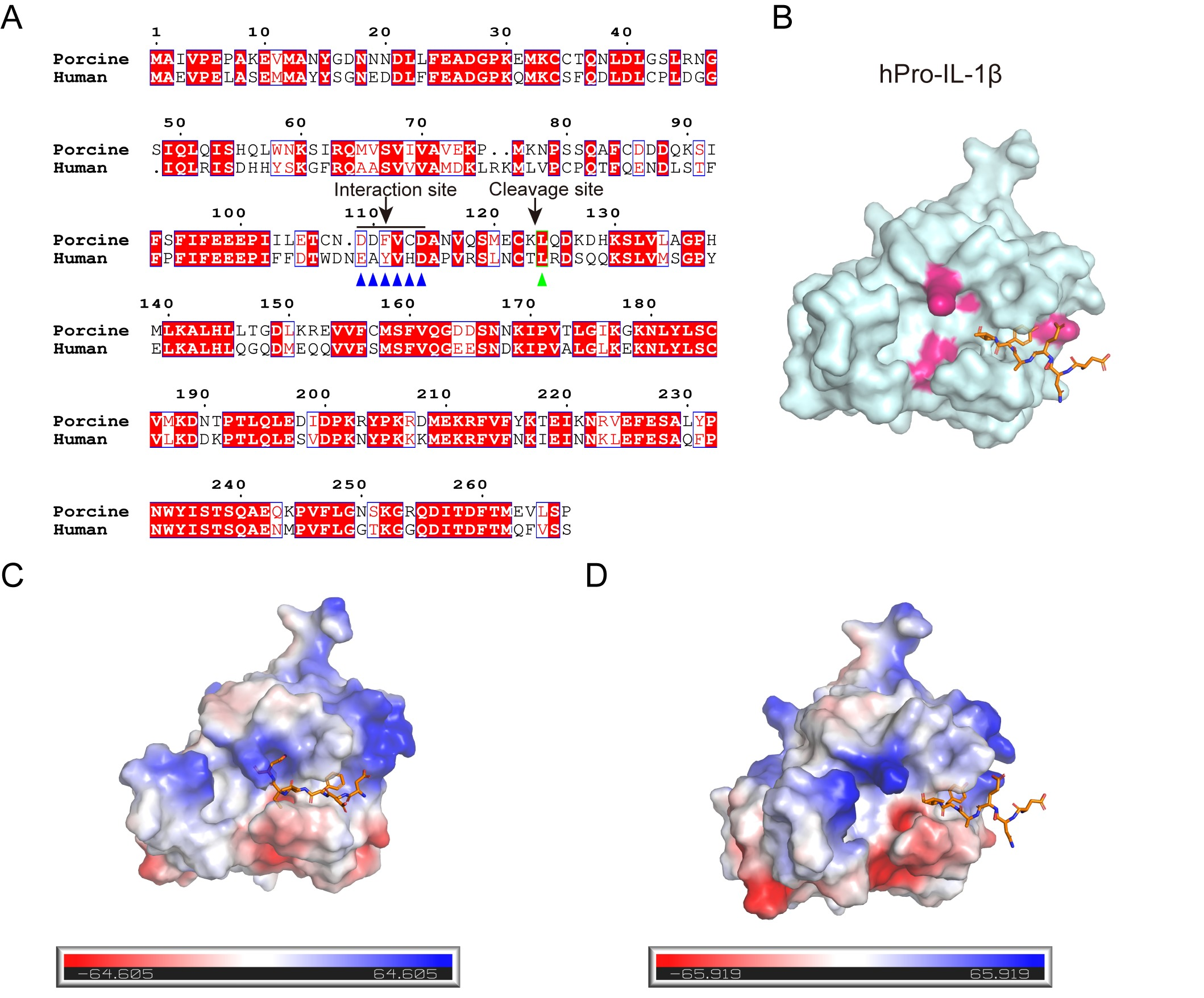

Supplement: S6 Fig — (A) Sequence alignment of the structural domains of sPro-IL-1β and hPro-IL-1β. Identical and similar amino acids are highlighted in red or white boxes, respectively. The alignment was performed using the ClustalOmega online tool. (B) The overall structure of SVV 3C is shown in surface representation with the 109-DNEAYV-114 motif of hPro-IL-1β shown in stick representation. Positively charged pocket (H48, R150, K157, H178 of SVV 3C) was marked in hotpink on surface model. (C and D) Analysis of the surface electrostatics of SVV 3C. The 109-DDFVCD-114 motif of sPro-IL-1β inserts into the positively charged pocket of SVV 3C (C), while the corresponding segment 109-DNEAYV-114 motif of hPro-IL-1β is distanced from the positively charged pocket of SVV 3C (D). Positively charged surface is colored blue and negatively charged surface red. The 109-DDFVCD-114 motif of sPro-IL-1β and 109-DNEAYV-114 motif of hPro-IL-1β are shown by the stick models. (TIF) [file ppat.1012398.s006.tif]
